# Evolutionarily recent, insertional fission of mitochondrial *cox2* into complementary genes in bilaterian Metazoa

**DOI:** 10.1186/s12864-017-3626-5

**Published:** 2017-03-31

**Authors:** Przemyslaw Szafranski

**Affiliations:** Department of Molecular and Human Genetics, Baylor College of Medicine, One Baylor Plaza, ABBR, R851C, Houston, TX 77030 USA

**Keywords:** Genome evolution, Mitochondrial gene fission, Horizontal gene transfer, Homing nuclease, *Campsomeris*, Hymenoptera

## Abstract

**Background:**

Mitochondrial genomes (mtDNA) of multicellular animals (Metazoa) with bilateral symmetry (Bilateria) are compact and usually carry 13 protein-coding genes for subunits of three respiratory complexes and ATP synthase. However, occasionally reported exceptions to this typical mtDNA organization prompted speculation that, as in protists and plants, some bilaterian mitogenomes may continue to lose their canonical genes, or may even acquire new genes. To shed more light on this phenomenon, a PCR-based screen was conducted to assess fast-evolving mtDNAs of apocritan Hymenoptera (Arthropoda, Insecta) for genomic rearrangements that might be associated with the modification of mitochondrial gene content.

**Results:**

Sequencing of segmental inversions, identified in the screen, revealed that the cytochrome oxidase subunit II gene (*cox2*) of *Campsomeris* (*Dielis*) (Scoliidae) was split into two genes coding for COXIIA and COXIIB. The COXII-derived complementary polypeptides apparently form a heterodimer, have reduced hydrophobicity compared with the majority of mitogenome-encoded COX subunits, and one of them, COXIIB, features increased content of Cys residues. Analogous *cox2* fragmentation is known only in two clades of protists (chlorophycean algae and alveolates), where it has been associated with piecewise relocation of this gene into the nucleus. In *Campsomeris* mtDNA, *cox2a* and *cox2b* loci are separated by a 3-kb large cluster of several antiparallel overlapping ORFs, one of which, *qnu*, seems to encode a nuclease that may have played a role in *cox2* fission.

**Conclusions:**

Although discontinuous mitochondrial protein genes encoding fragmented, complementary polypeptides are known in protists and some plants, split *cox2* of *Campsomeris* is the first case of such a gene arrangement found in animals. The reported data also indicate that bilaterian animal mitogenomes may be carrying lineage-specific genes more often than previously thought, and suggest a homing endonuclease-based mechanism for insertional mitochondrial gene fission.

**Electronic supplementary material:**

The online version of this article (doi:10.1186/s12864-017-3626-5) contains supplementary material, which is available to authorized users.

## Background

Mitochondria contain residual genomes (mtDNA) with the majority of their original α-proteobacterial gene set transferred to the host nucleus or lost by other means [1, 2]. Among the most compact mitogenomes are those of multicellular animals (Metazoa) with bilateral symmetry (Bilateria) [3–7]. They usually carry 37 annotated intron-less genes, of which only 13 are protein-coding, and they have dramatically reduced or entirely absent intergenic regions. Deviations from this conserved gene set in the Bilateria are rare and comprise mostly tRNA genes. Although cases of a protein-coding gene missing from mtDNA are known in Vertebrata (*atp8*, *nad5*, *nad6*), Chaetognatha (*atp6*, *atp8*), Nematoda (*atp8*), and Platyzoa (*atp8*) [6], some of them may actually represent the presence of highly derived gene variants rather than true gene loss [6, 8, 9]. The only lineage-specific translated genes identified in bilaterian mitogenomes are the f- and m-ORFs found in bivalves (Mollusca) with doubly uniparental inheritance of mitochondria [10, 11]. Moreover, a conserved non-overlapping ORF was identified in the control region (CR) of mammalian mtDNA [12], unassigned ORF sequences have been found in *Lingula* (Brachiopoda) [13], and an ORF that likely originated through the duplication of a canonical gene was found in the mtDNA of oysters (Mollusca) [14].

The association of cases of presumptive mitochondrial gene loss or acquisition of new ORFs with the increase of rate of nucleotide substitutions and mtDNA rearrangements prompted speculation that modifications of the mitochondrial gene content in Bilateria might be more common than is currently assumed due, in part, to the relative underrepresentation of faster-evolving mitogenomes among sequenced mtDNAs. Indirectly supporting this hypothesis, additional protein-coding genes have been identified in the mtDNA of basal metazoans [6], and the transfer of genetic material from the mitochondria to the nucleus is a well-known phenomenon that still occurs in almost all eukaryotes, although it usually generates nuclear pseudogene copies of mitochondrial genes (NUMTS) [15–17]. Functional relocation of mitochondrial genes to the nucleus, where they would resume their expression thus allowing for the loss of their mitochondrial copy, has been shown to continue in protists and plants, and involve both intact and fragmented genes [18]. Interestingly, half of the split, originally mitochondrial genes have at least one of the derived genes transferred to the nucleus and lost from the mtDNA. They include (i) *cox1* in the majority of eukaryotic supergroups (excluding, among others, plants and Opisthoconta) where it split at the 3’ end and the 3’ terminal fragment was transferred to the nucleus [19]; (ii) *cox2* in Alveolata and chlorophycean algae (Chlorophyta), where both or only the 3’ terminal half of the gene was transferred to the nucleus [20–24]; (iii) *rpl2* in eudicots (Angiospermae), where both or only the 5’ or 3’ section was transferred to the nucleus [25]; and (iv) *sdhB* in Euglenozoa, where both derived genes were transferred to the nuclear genome [26]. The fission of mitochondrial genes for proteins with transmembrane topology, which might be difficult to transfer to mitochondria if they were encoded in the nucleus, may allow for their partial relocation limited to a region coding for a less hydrophobic part of a protein [19–21, 24, 27–29]. Split protein-coding genes with both derived genes residing in mtDNA include *nad1*, *nad2* and *rps3* of ciliates (Alveolata) [30–32] and *ccmF*_*N*_ and *ccmF*_*C*_ (*ccb*) orthologs of bacterial *ccmF* (*ccl1*) in plants such as *Marchantia* and several groups of angiosperms [25, 33].

Here, the correlation between mitochondrial gene loss, gene fragmentation or the addition of new genes and an increased rate of mtDNA evolution was explored using the mitogenomes of the apocritan Hymenoptera (Arthropoda, Insecta). Hymenoptera in the suborder Apocrita, which includes Aculeata and families grouped in the paraphyletic “Parasitica”, were selected for the present studies due to more rapid evolution of their mtDNA compared with the evolution of the majority of sequenced mitogenomes of other insects and metazoans in general [34–36]. The screen applied in these studies retrieved unique for animals fission of a canonical mitochondrial gene, *cox2*, in representatives of *Campsomeris* (*Dielis*) (Scoliidae). Scoliids are a family of solitary wasps that develop as idiobiont ectoparasitoids of the larval stages of Scarabaeoidea and less often other Coleoptera. *Cox2* encodes the subunit II of cytochrome c (CytC) oxidase (COX) that mediates the transfer of electrons from CytC to COX subunit I (COXI) during oxidative phosphorylation (OXPHOS). The split of *cox2* in two genes for complementary COXIIA and COXIIB polypeptides likely occurred through intragenic insertion of a cluster of several ORFs, one of which encodes a putative endonuclease that might have been directly involved in the process of *cox2* fission.

## Results

### Mitochondrial *cox2* gene in *Campsomeris* is split in half

**Fig. 1:**
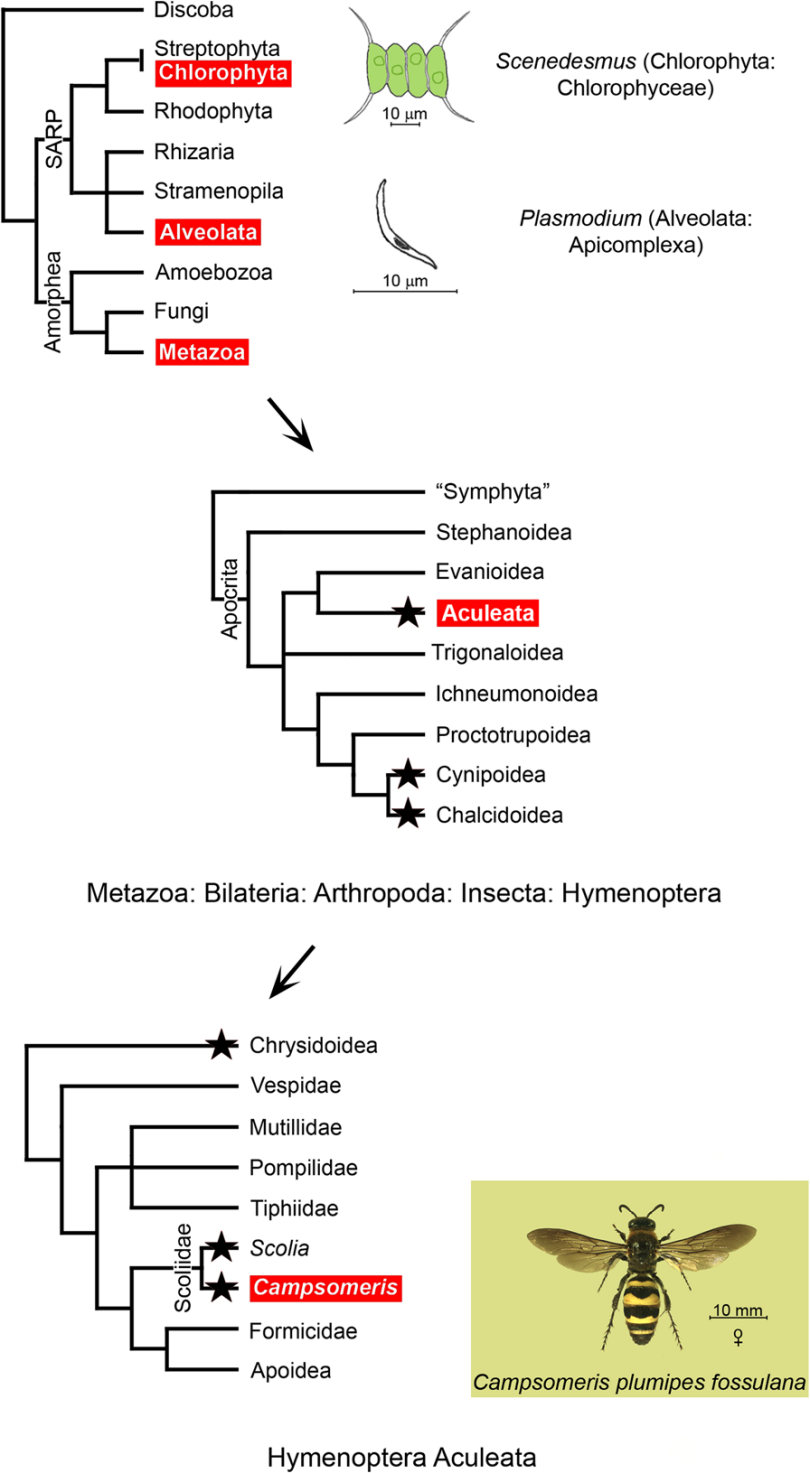
*Cox2* fission across phylogeny. Lineages harboring taxons carrying the *cox2* gene split into derived genes are marked in red. Asterisks denote the presence of segmental inversions of *cox1* versus *rrnL* in the mtDNA of Hymenoptera (Additional file 1: Figure S1). Simplified tree topologies are based on recent revisions by He et al. [70] (Eukaryota), Mao et al. [71] (Hymenoptera) and Johnson et al. [72] (Aculeata)

**Fig. 2:**
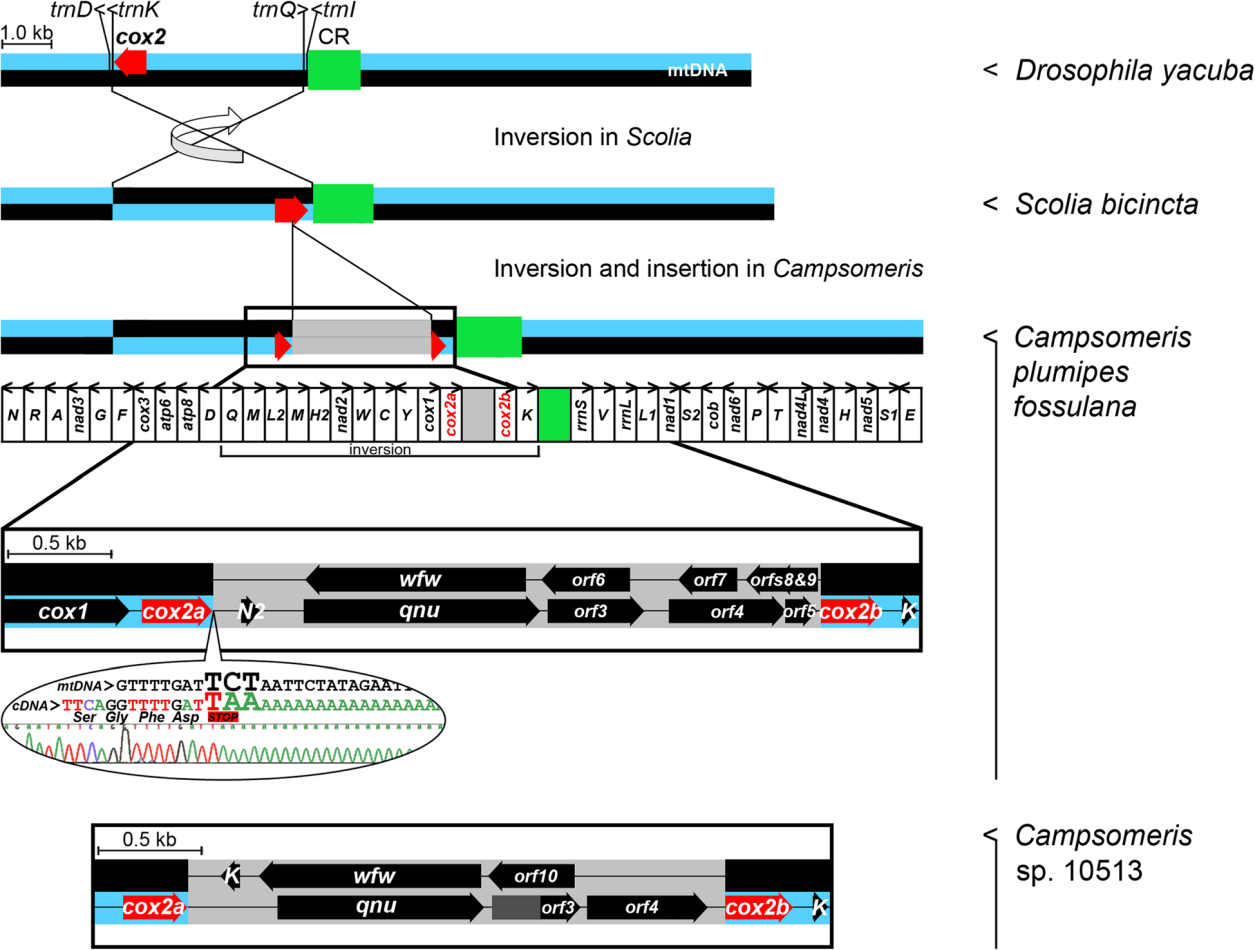
Split of the *cox2* gene in mtDNA of *Campsomeris*. Circular mitogenomes are arbitrarily linearized at the 3’ end of *trnN*. The *Drosophila* mtDNA represents the plesiomorphic mitogenome of Pancrustacea. The inverted mtDNA segment in *Scolia* (KT276222) and *Campsomeris* (KT740996, KX090217) is flanked by the *trnD* gene and the control region (CR). The inversion might have occurred due to recombination between similar and oppositely oriented *trnK* and *trnQ* genes. Other modifications of the *C. p. fossulana* mitogenome include single-gene inversions of *trnQ*, *trnC* and *trnS1*, translocations of *trnF* and *trnL2*, shuffling of *trnS1*, duplication of *trnM*, the presence of *trnH* ^*CAT*^ in addition to *trnH* ^*CAC*^, the presence of *trnK* ^*AAA*^ (within the CR) in addition to *trnK* ^*AAG*^, and the loss of *trnI* (or its replacement by a putative *trnI* gene located within *rrnL*). *TrnS1*, *trnR* and putative *trnI* encode tRNAs that lack the TψC (T) arm. The chromatopherogram of the cDNA sequence corresponding to the *cox2a* mRNA 3’ end (RACE product) shows that the *cox2a* stop codon, UAA, is generated by the polyadenylation of U (corresponding to T^6195^; KT740996). The positions of genes/ORFs in *Campsomeris* sp. HA10513 were deduced from comparison with the corresponding regions of *C. p. fossulana* mtDNA (5’ part of the reading frame of *Campsomeris* sp. HA10513 *orf3*, marked dark gray, is shifted in comparison to the reading frame of *C. p. fossulana orf3*). *C. p. fossulana orf3-9* correspond to polyadenylated mRNAs that have been mapped by RACE. *H2*, *N2*, and *K* denote *trnH* ^*CAT*^, putative (low covariance score) *trnN* ^*AAT*^ and *trnK* ^*AAA*^ (*Campsomeris* sp. HA10513) or *trnK* ^*AAG*^ (*C. p. fossulana*), respectively

The exploration of hymenopteran mitogenomes for potential changes in gene content was guided by a PCR-based screen that primarily targeted mtDNA segmental inversions, as well as deletions and duplications/insertions. Large, multigenic inversions represent uncommon type of mitochondrial genome rearrangements and can be mechanistically linked to gene translocation, fragmentation, loss and duplication, or the acquisition of new genes. For instance, both inversions and modifications of gene structure and content may arise during the process of the repair of DNA double-stranded breaks by nonhomologous end-joining [37]. The screen was designed to identify inversions of *cox1-* versus *rrnL*-bearing segments of mtDNA (Additional file 1: Figure S1). In the typical circular mitogenome of insects and other pancrustaceans [38], *cox1* and *rrnL* genes are separated from one another by two and three rearrangement hotspots located within clusters of tRNA genes (*trn[I-Q-M]*; *trn[W-C-Y]*; *cox1*; *trn[K-D]*; *trn[A-R-N-S-E-F]*; *trn[T-P]*; *rrnL*) [34]. The primers used in the screen were designed to map within conserved regions of *cox1* and *rrnL*, and their orientation permitted the amplification of mtDNA between the two genes if one of the genes was inverted. This strategy led to the identification of *cox1* versus *rrnL* segmental inversions in representatives of Scoliidae and Chrysididae of Aculeata and in Cynipidae and Chalcidoidea of Proctotrupomorpha (Table 1, Fig. 1). Subsequent sequencing of these rearranged mitogenomes revealed the presence of a 3.0-kb insertion within the *cox2* gene of *Campsomeris* (*Dielis*) *plumipes* (Drury) (ssp. *fossulana* (Fab.)) of Scoliidae (Figs. 1 and 2). Thereafter, a similar 2.6-kb insertion was found in *cox2* of another yet undescribed *Campsomeris* (*Dielis*) sp. HA10513 (Fig. 2).

**Table 1:** Systematic list of hymenopteran species analyzed for the presence of *cox1* versus *rrnL* segmental inversions in their mtDNA

Species	Taxonomy	Inversion of *cox1* vs *rrnL*
	“Symphyta”	
*Cephus cinctus* Norton	Phytophagous groups: Cephoidea: Cephidae	-
*Orussus occidentalis* (Cresson)	Orussoidea: Orussidae	-
	Apocrita: “Evaniomorpha”	
*Schlettererius cinctipes* (Cresson)	Stephanoidea: Stephanidae	-
*Evania appendigaster* (L.)	Evanioidea: Evaniidae	-
*Pseudogonalos hahnii* (Spinola)	Trigonaloidea: Trigonalidae	-
	Apocrita: Ichneumonomorpha & Proctotrupomorpha	
*Cotesia vestalis* (Haliday)	Ichneumonoidea: Braconidae: Microgastrinae	-
*Spathius agrili* Yang	Ichneumonoidea: Braconidae: Doryctinae	-
*Diachasmimorpha longicaudata* (Ashmead)	Ichneumonoidea: Braconidae: Opiinae	-
*Macrocentrus camphoraphilus* He & Chen	Ichneumonoidea: Braconidae: Macrocentrinae	-
*Aphidius gifuensis* Ashmead	Ichneumonoidea: Braconidae: Aphidiinae	-
*Protichneumon grandis* (Brulle)	Ichneumonoidea: Ichneumonidae: Ichneumoninae	-
*Diadegma semiclausum* (Hellén)	Ichneumonoidea: Ichneumonidae: Campopleginae	-
*Enicospilus* sp.	Ichneumonoidea: Ichneumonidae: Ophioninae	-
*Vanhornia eucnemidarum* Crawford	Proctotrupoidea: Vanhorniidae	-
*Cynips quercusfolii* L.	Cynipoidea: Cynipidae: Cynipini	inv
*Synergus* sp.	Cynipoidea: Cynipidae: Synergini	inv
*Philotrypesis pilosa* Mayr	Chalcidoidea: Agaonidae	inv
*Torymus auratus* (Müller)	Chalcidoidea: Torymidae	inv
	Apocrita: Aculeata	
*Primeuchroeus* sp.	Chrysidoidea: Chrysididae: Chrysidinae	inv
*Chrysis* sp.	Chrysidoidea: Chrysididae: Chrysidinae	inv
*Polistes carolina* (L.)	Vespoidea: Vespidae: Polistinae	-
*Abispa ephippium* (Fab.)	Vespoidea: Vespidae: Eumeninae	-
*Euodynerus* sp.	Vespoidea: Vespidae: Eumeninae	-
*Wallacidia oculata* (Fab.)	Vespoidea: Mutillidae: Mutillinae	-
*Ceropales femoralis* Cresson	Vespoidea: Pompilidae: Ceropalinae	-
*Pepsis elegans* L.	Vespoidea: Pompilidae: Pepsinae	-
*Anoplius lepidus atramentarius* (Dahl.)	Vespoidea: Pompilidae: Pompilinae	-
*Myzinum maculatum* (Fab.)	Vespoidea: Tiphiidae: Myzininae	-
*Campsomeris plumipes fossulana* (Fab.)	Vespoidea: Scoliidae: Campsomerinae	inv
*Campsomeris* sp. HA10513	Vespoidea: Scoliidae: Campsomerinae	inv
*Scolia bicincta* Fab.	Vespoidea: Scoliidae: Scoliinae	inv
*Scolia dubia* Say	Vespoidea: Scoliidae: Scoliinae	inv
*Solenopsis geminata* (Fab.)	Vespoidea: Formicidae: Myrmicinae	-
*Apis mellifera* L.	Apoidea: Apidae: Apinae	-
*Bombus ignitus* Smith	Apoidea: Apidae: Bombinae	-
*Melipona bicolor* (Lep.)	Apoidea: Apidae: Meliponinae	-
*Sceliphron caementarium* (Drury)	Apoidea: Sphecidae: Sceliphrinae	-
*Ammophila* sp.	Apoidea: Sphecidae: Ammophilinae	-
*Sphex pensylvanicus* L.	Apoidea: Sphecidae: Sphecinae	-
*Bicyrtes quadrifasciata* (Say)	Apoidea: Crabronidae: Bembicinae	-
*Cerceris* sp.	Apoidea: Crabronidae: Philanthinae	-

To determine the incidence of the *cox2* split within Scoliidae and verify its confinement to this aculeate family, the mtDNA of two *Scolia* species, *S. bicincta* and *S. dubia* (Scoliidae), and *cox2* of randomly selected representatives of hymenopteran families of Tiphiidae, Mutillidae, Pompilidae, Formicidae, and Apoidea (Table 1), which are phylogenetically more closely related to Scoliidae, were sequenced. The integrity of the *cox2* gene was preserved in all the additionally analyzed Hymenoptera, suggesting that *cox2* fission may be confined to *Campsomeris* or Campsomerinae. Of note, the mitogenomes of *Scolia* also featured segmental inversion corresponding to the inversion of *trn[Q-M-L2-M-H]-nad2-trn[W-C-Y]-cox1-cox2a*-insert-*cox*2*b-trnK* found in *Campsomeris* mtDNA (Fig. 2).

### Mature *cox2a* and *cox2b* transcripts are discrete and polyadenylated

The *cox2*-splitting insertion occurred within a relatively less conserved region of the gene (Additional file 1: Figure S2). It divided *cox2* into *cox2a*, encoding two transmembrane helices, the N-terminal intermembrane space domain and the “heme-patch” region (containing Trp^105^, which functions as the point of electron entry from CytC; KT740996) of the canonical COXII, and *cox2b*, encoding intermembrane space C-terminal half of COXII containing the binuclear Cu_A_ center. In *C. p. fossulana*, the insertion is located in-frame with *cox2a* and *cox2b*, meaning that *cox2* might still be expressed as a single polypeptide that is larger than the original one. In-frame insertions in the corresponding region of *cox2* in ciliates, brown algae, microflagellata, and bacteria resulted in enlargement, not fission, of *cox2* genes [39, 40].

To determine whether *C. p. fossulana* COXII is encoded by an enlarged, single *cox2* gene or separate *cox2a* and *cox2b* genes, the 5’ and 3’ ends of the *cox2* transcripts were mapped by RACE. This analysis showed that *cox2a* and *cox2b* transcripts are discrete, non-overlapping, and polyadenylated. It also showed that the *cox2a* termination codon, UAA, was completed by polyadenylation (Fig. 2). Moreover, RACE analysis of the *cox2* transcripts did not provide evidence for *cox2* splicing in *Campsomeris*. However, since a group II intron is present within the *cox1* gene in the mitogenomes of Annelida (the only known case of mitochondrial RNA splicing in Bilateria) [41], the absence of residual *cox2* pre-mRNA splicing was additionally verified by PCR using *cox2a*- and *cox2b*-specific primers corresponding to sequences flanking the inserted DNA. The PCR did amplify a 3-kb DNA product from *Campsomeris* mtDNA but did not amplify any product from the cDNA, again arguing against even residual *cox2* RNA splicing or *cox2a* and *cox2b* RNA trans-splicing into a single mRNA.

The relative levels of *cox2a* and *cox2b* transcripts were determined by RT-qPCR and appeared to differ from one another. In comparison with the RNA level of *cox1*, the *cox2a* transcript was slightly less abundant whereas the *cox2b* transcript was present at a level approximately 3 times higher. This finding further supports the results obtained by RACE that mature *cox2a* and *cox2b* mRNAs represent separate entities.

### *Cox2a* and *cox2b* genes are translated

**Fig. 3:**
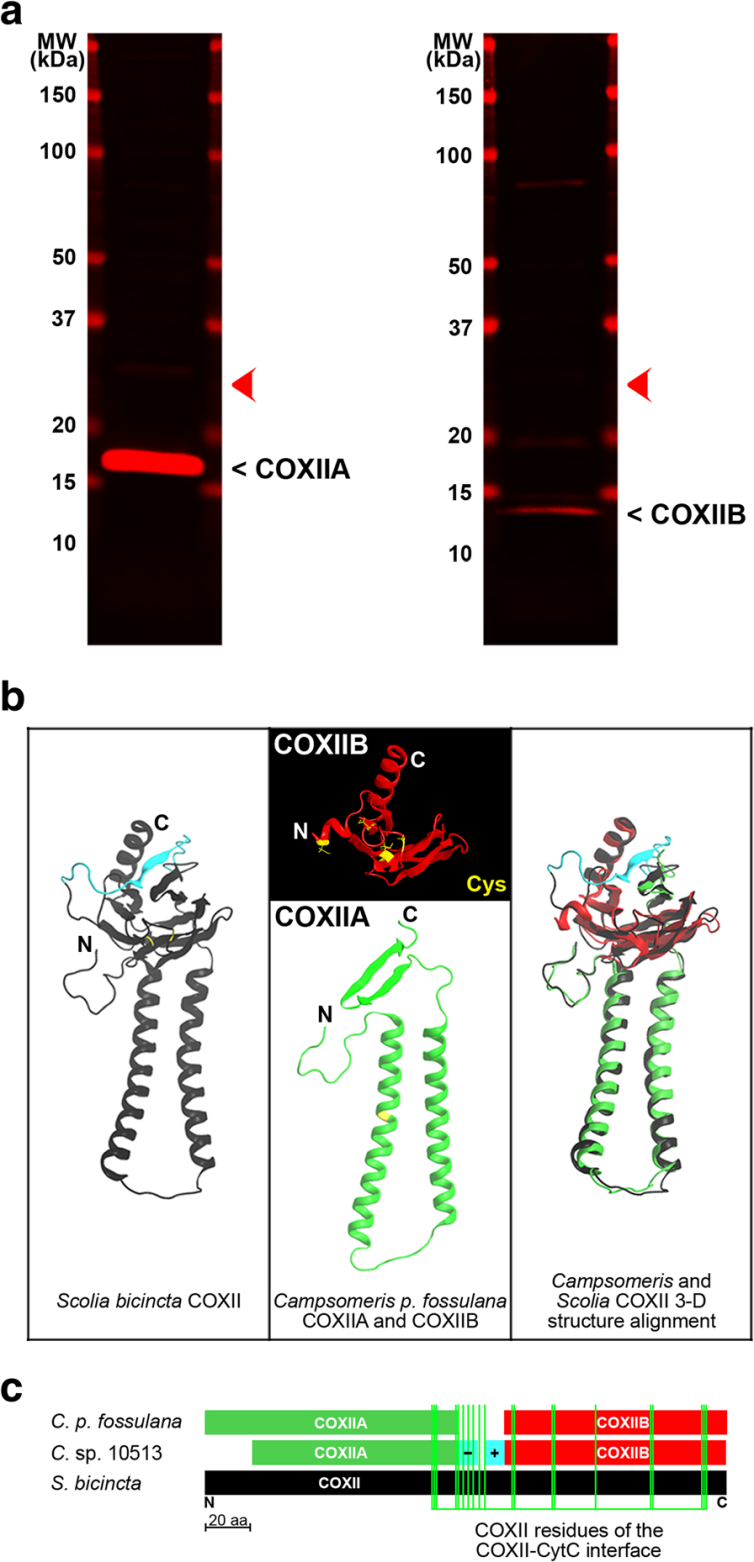
The heterodimeric structure of *Campsomeris* COXII. **a** Western blot analysis of *C. p. fossulana* COXIIA and COXIIB polypeptides. The deduced position of the COXII band of *Scolia*, corresponding to that of other bilaterian animals with the exception of *Campsomeris*, is indicated by red arrowheads. **b** Tertiary structures of *C. p. fossulana* and *S. bicincta* COXII polypeptides, modelled in I-TASSER using crystal structures of bovine, *Paracoccus denitrificans* and *Rhodobacter sphaeroides* COXII (PDB: 1oczB, 3hb3B and 1m56B, respectively) as templates. The structural similarity between split COXII of *C. p. fossulana* and intact COXII of *S. bicincta* is shown in the superimposition panel. The central part of COXII of *Scolia* (and other pancrustaceans), which is missing in *C. p. fossulana* COXIIA and COXIIB, is depicted in blue. **c** Schematic alignment of COXII polypeptides and some of the regions proposed to be involved in COXII heterodimer reassembly. Terminal domains that are likely engaged in electrostatic interactions are shown in blue and marked “-“and “+”, respectively. The COXII/CytC interface is defined as in Schmidt et al*.* [49]

To determine whether *C. p. fossulana cox2a* and *cox2b* genes did not represent transcribed pseudogenes, the *C. p. fossulana* mitochondrial proteome was analyzed by western blotting using polyclonal antibodies (Abs) generated against deduced COXIIA and COXIIB synthetic epitopes. Western blot analysis revealed that *Campsomeris cox2* was translated as two separate polypeptides, COXIIA and COXIIB, with sizes comparable to those predicted from the cDNA sequences (115 and 100 amino acids, respectively) (Fig. 3a, Additional file 1: Figures S3 and S4). Moreover, none of the Abs detected a larger polypeptide that might otherwise indicate the occurrence of posttranslational, intein-mediated trans-splicing of COXIIA and COXIIB. To date, split *cox2* genes have only been found in two groups of protists, i.e. Chlorophyta [20–22, 24] and Alveolata [23, 42, 43] (Fig. 1). Alignment of the predicted sequences of the COXII split sites indicated that, in protists, *cox2* splitting occurred in the position corresponding to the COXII splitting site in *Campsomeris* (Additional file 1: Figure S2). In-frame insertions into *cox2* in ciliates (Alveolata), which generated enlarged COXII polypeptides, also occurred in the same position as COXII-splitting insertions in other protists and *Campsomeris*.

The three-dimensional structures of *C. p. fossulana* COXIIA and COXIIB polypeptides were modelled using a template-based method with the I-TASSER algorithm (NovaFold) (Fig. 3b). When superimposed on a similarly determined structure of the intact COXII of *S. bicincta*, both COXIIA and COXIIB showed a good fit supporting their functionality (Fig. 3b). COXIIA and COXIIB of Chlorophyceae (Chlorophyta) and Alveolata have been proposed to reassemble into functional heterodimeric COXII by taking advantage of the interactions between their unique C- and N-terminal extensions, respectively [22, 23]. Sequencing of the ends of *C. p. fossulana cox2a* and *cox2b* cDNAs indicated that *C. p. fossulana* COXIIA and COXIIB do not have extended terminal regions (Additional file 1: Figures S3 and S4), and Instead, they might reassemble by taking advantage mostly of shape and electrostatic internal complementarity. Reconstitution of active proteins even from multiple fragments, including those with breakpoints mapping within well-defined functional domains, has been demonstrated for numerous proteins [44–46]. Moreover, by analogy to intramolecular interactions found in *Paracoccus denitrificans* COXII [47], the N-terminal loop of COXIIA might contribute to COXII heterodimer assembly by interacting in the mitochondrial intermembrane space with COXIIB. Interestingly, the N-terminal intermembrane space domain of *Campsomeris* sp. HA10513 COXIIA is shortened, but in this case, the N-terminus of COXIIB is significantly enriched in positively charged Lys residues (Additional file 1: Figure S2). Since the C-terminus of COXIIA contains negatively charged Glu residues (Additional file 1: Figure S2), the COXII heterodimer might be additionally stabilized in this case by a salt bridge between the C- and N-termini of COXIIA and COXIIB, respectively (Fig. 3c). Finally, the involvement of interacting proteins usually dramatically improves the kinetics of split protein reassembly [48]. COXII, together with COXI and COXIII, form the catalytic core of respiratory complex IV, surrounded by several COX subunits that are imported from the cytosol. Some of these proteins likely interact with COXIIA and COXIIB, contributing to the assembly of the functional COXII heterodimer. Of note, COXII splitting occurred within the CytC binding interface, the amino acid residues of which are scattered through the entire COXIIB and C-terminal intermembrane space region of COXIIA (Fig. 3c) [49]. Thus, COXII local folding around its binuclear center might be further adjusted during interactions with CytC.

### Hydrophobicity and Cys content of COXIIA and COXIIB

Comparison of the amino acid content of *Campsomeris* COXIIA and COXIIB, with that of an intact COXII of *S. bicincta* and *Apis mellifera* revealed a decrease in fragmented COXII of Ile residues (the most abundant amino acid residue in COXII) and an increase of Cys residues (Additional file 1: Figure S5).

**Fig. 4:**
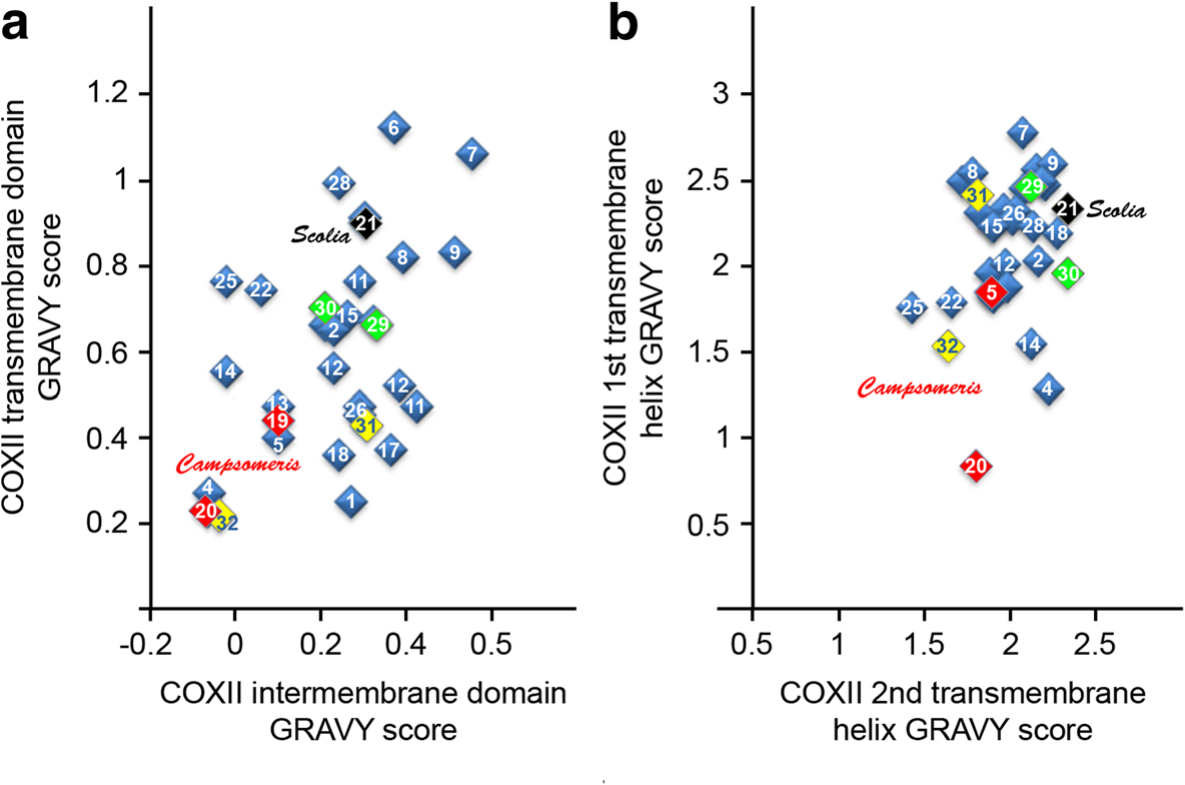
Relative hydrophobicity of hymenopteran COXII polypeptides. The grand average of hydropathy (GRAVY) values were estimated using the GRAVY Calculator (http://www.gravy-calculator.de/; Kyte and Doolittle hydrophobicity scale). GRAVY values are plotted in red and black for *Campsomeris* species and *S. bicincta*, respectively; in green for COXII of the non-hymenopteran species, *Pediculus* (“Phthiraptera”) (29) and *Drosophila* (Diptera) (30); and in yellow for COXII of chlorophycean algae *Scenedesmus* (31) and *Chlamydomonas* (32). The following genera of Hymenoptera were taken into account: 1, *Perga*; 2, *Cephus*; 3, *Orussus*; 4, *Schlettererius*; 5, *Evania*; 6, *Cotesia*; 7, *Phanerotoma*; 8, *Spathius*; 9, *Diachasmimorpha*; 10, *Macrocentrotus*; 11, *Aphidius*; 12, *Diadema*; 13, *Enicospilus*; 14, *Vanhornia*; 15, *Nasonia*; 16, *Philotripesis*; 17, *Wallacidia*; 18, *Cephalonomia*; 19, 20, *Campsomeris* (*p. fossulana* and sp. HA10513, respectively); 21, *Scolia*; 22, *Apis*; 23, *Bombus*; 24, *Melipona*; 25, *Solenopsis*; 26, *Polistes*; 27, *Abispa*; 28, *Primeuchroeus*. The transmembrane and intermembrane space domains of COXII correspond to *Campsomeris* COXIIA and COXIIB polypeptides, respectively. The transmembrane regions were predicted using the TMHMM method (http://www.cbs.dtu.dk/services/TMHMM/) [73]. **a** The split COXII of *Campsomeris* is twice less hydrophobic than its intact counterpart in the next most closely related *Scolia* and is among the least hydrophobic eukaryotic COXII polypeptides. **b** The hydrophobicity of the first transmembrane helix of *Campsomeris* COXIIA is among the lowest in eukaryotes

The impact of the reduced presence of hydrophobic Ile as well as Leu on the overall character of *Campsomeris* COXIIA and COXIIB was estimated by calculating the average hydropathy (GRAVY) for COXIIA, for the first and second transmembrane helices of COXIIA, for COXIIB, and for the corresponding regions of intact COXII of other Hymenoptera and representatives of other taxonomic groups. A comparison of the GRAVY values showed that *Campsomeris* COXIIA and, to a lesser degree, COXIIB exhibited reduced hydrophobicity compared with the corresponding regions of COXII in *Scolia* and in the majority of other Hymenoptera (Fig. 4a). The hydrophobicity of the first transmembrane helix of *Campsomeris* COXIIA was also among the lowest in Hymenoptera (Fig. 4a). Interestingly, the hydrophobicity of *Campsomeris* COXIIA and COXIIB polypeptides was similar to that of *Chlamydomonas* COXIIA and COXIIB or *Scenedesmus* COXIIB, all of which are encoded in the nuclear genome and transported to mitochondria.

**Fig. 5:**
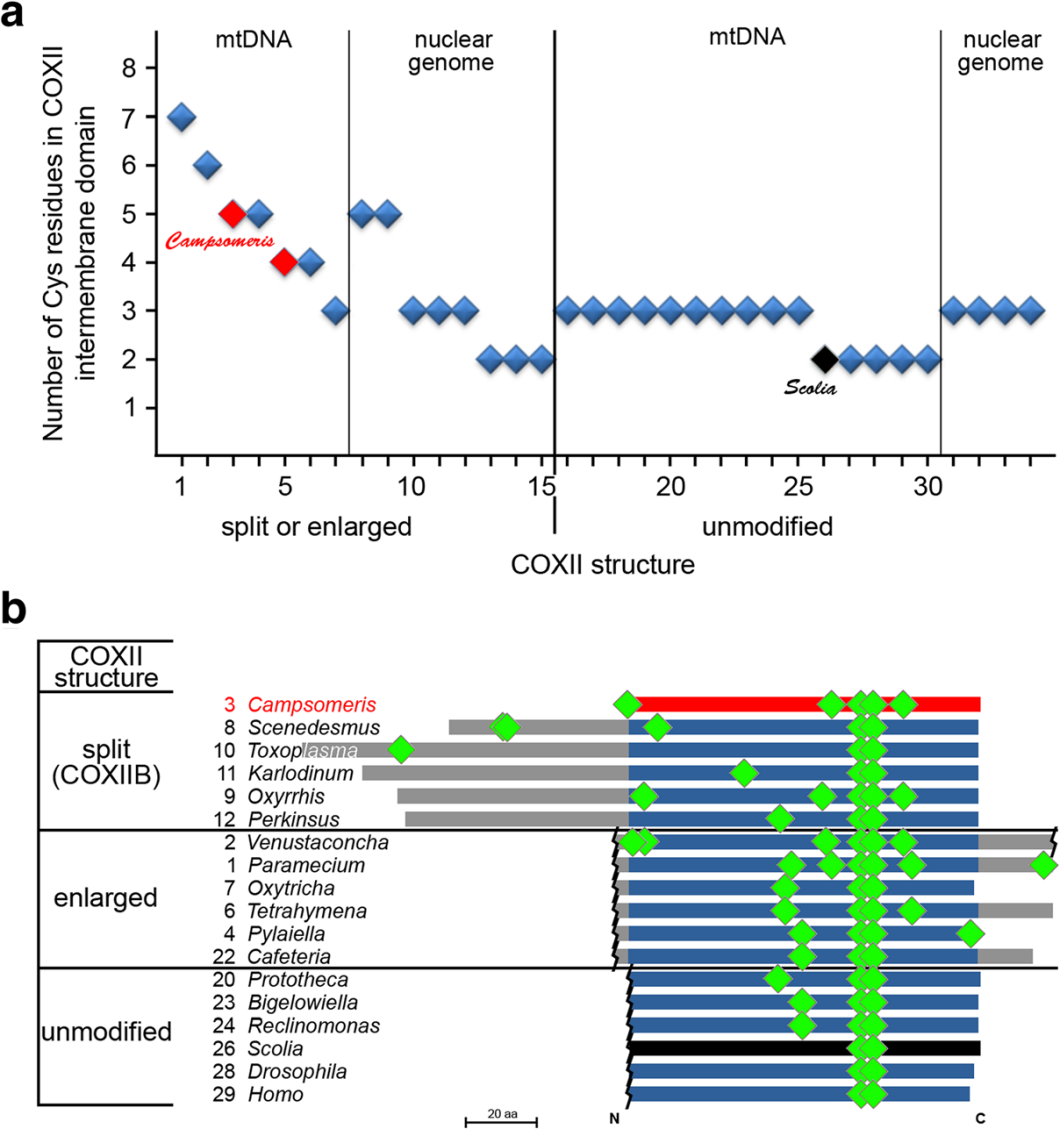
Cys residue enrichment of derived COXII polypeptides. **a** Correlation between split or enlargement of COXII and Cys content of the COXII domain exposed to the mitochondrial intermembrane space, equivalent to COXIIB. Data for *Campsomeris* species (*p. fossulana* (3) and sp. HA10513 (5)), and *S. bicincta* (26) are plotted in red and black, respectively. Split or enlarged polypeptides encoded by mtDNA contained an average of two-fold or more Cys residues than unmodified COXII polypeptides (Mann-Whitney *U*-test: *P* = 0.001). A complete list of genera and the taxonomy of analyzed organisms are shown in (Additional file 1: Table S1). **b** The distribution of Cys residues (*green marks*) along the COXII intermembrane space domain (*blue or black* (*Scolia*)) homologous to *Campsomeris* COXIIB (*red*)

Cys residues are the only reactive amino acid side chains with substantially changed representation in *Campsomeris* COXIIB compared with intact COXIIs (Additional file 1: Figure S5). A phylogeny-wide survey of the Cys content in the COXII intermembrane domain, corresponding to COXIIB, revealed that this domain was specifically enriched in Cys not only in *Campsomeris* COXIIB but also in other split or enlarged COXII polypeptides (Fig. 5), all of which might benefit from redox-based assistance to maintain their proper folding or intermolecular interactions.

### *Cox2a* and *cox2b* loci are separated by a cluster of antiparallel overlapping transcribed ORFs

Sequencing of the *C. p. fossulana* 3-kb insert and its conceptual translation revealed, in addition to the mentioned continuous ORF bridging *cox2a* and *cox2b*, the presence of five ORFs on the complementary mtDNA strand, ranging in size from 0.2 to 1.1 kb (Fig. 2). RACE analysis of *C. p. fossulana* mitochondrial cDNA indicated that all ORFs were transcribed and their RNAs were polyadenylated, with﻿ cleavage/polyadenylation sites being much more scattered along the transcripts than in case of canonical mitochondrial genes. This analysis also revealed that the continuous ORF, including *cox2a* and *cox2b*, was transcribed as RNA that was processed into *cox2a* and *cox2b* mRNAs and other mRNAs, four of which (*qnu* and *﻿orfs3-5*) had in-frame TAA termination codons generated by polyadenylation (Fig. 2). In *Campsomeris* sp. HA10513, continuity of the ORF corresponding to the *C. p. fossulana* largest ORF (including *cox2a* and *cox2b*) was interrupted in the middle of the insert, and there were only two ORFs, *wfw* and *orf10*,﻿ on the opposite to *cox2* strand (*orf10* di﻿d ﻿not share amino acid sequence similarity with polypeptides deduced from any of the *C. p. fossulana* ORFs) (Fig. 2). Pairwise alignments of deduced amino acid sequences of the inserted ORFs from the two *Campsomeris* species identified four groups of ORFs, *qnu*, *wfw*, *orf3* and *orf4*, with orthologous ORFs sharing extensive similarity and hence being likely of potentially functional significance.

Nucleotide and protein database searches using BLAST revealed that none of the ORFs encoded by the inserted DNA fragment had significant sequence similarity at the DNA or protein level to previously described genes, thus obscuring the origin of the insertion (*qnu* exhibits limited stretches of sequence similarity that are discussed in the next section). The A + T content of the inserted DNA fragment was ~13% lower compared to that of the remaining part of the *C. p. fossulana* mitogenome (Additional file 1: Figure S6), and was reflected by the decreased frequency of almost half of the A- and T-containing synonymous codons of the inserted ORFs (Additional file 1: Table S2).

A very distinctive feature of the insert was the antiparallel overlap of its ORFs (Fig. 2). *Cis*-natural sense antisense transcripts (*cis*-NATs) are found relatively frequently, even in the genomes of higher eukaryotes [50, 51]. However, extensive bidirectional overlapping is rare especially among protein-coding genes because sequence variants in one gene can often have deleterious effects on the sequence of the complementary gene. In mitochondria, such gene arrangement has been proposed for *cox1* and putative gene *gau* [52]. It seems interesting in this context that the open reading frames of overlapping *qnu* and *wfw*, as well as *orf3* (to a lesser extent) and *orf4*, have been preserved despite experiencing numerous indels as was visualized by a pairwise comparison of their sequences from two *Campsomeris* species (Additional file 1: Figure S7).

**Fig. 6:**
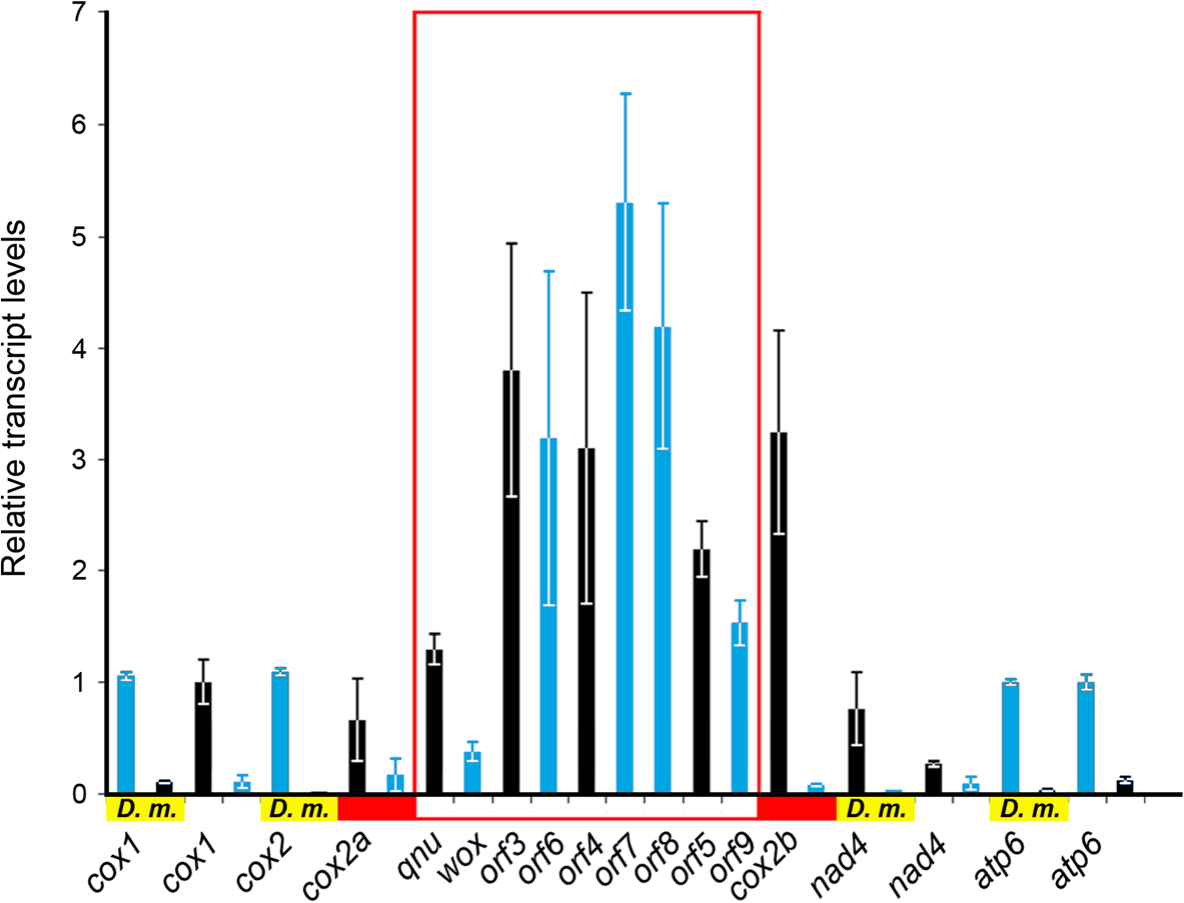
Transcript levels of selected *C. p. fossulana* and *D. melanogaster* mitochondrial genes. Copy numbers of the analyzed genes were confirmed by qPCR to be mutually equal to each other within a species. Levels of transcripts from mtDNA strands corresponding to the majority and minority strands of the insect ancestral mitogenome are marked in blue and black, respectively. Transcript levels of *C. p. fossulana* inserted genes are shown within the red frame and, in most cases, surpass those of the canonical mitochondrial genes. Abbreviation: *D.m*., *D. melanogaster*

RT-qPCR-determined relative transcript levels of the inserted genes were in most cases 2-3 times higher than those of canonical mtDNA-encoded genes (Fig. 6). For each inserted pair of antiparallel overlapping genes, with the exception of *qnu*-*wfw*, both transcripts were present at relatively higher levels. In contrast, transcripts that were antisense to the canonical mitochondrial genes, were usually present at low levels, resembling mRNA profiles of *Drosophila* (Fig. 6) and human mitochondria [53]. Higher levels of *cis*-NATs versus non-*cis*-NATs have also been found in mammalian [50] and *Arabidopsis* [54] transcriptomes. In *Campsomeris*, the increase in RNA levels of some transcripts might indicate their mixed origin from the mitochondria and nucleus. No evidence of heteroplasmy was detected by sequencing RACE products corresponding to the inserted ORFs, *cox2a* and *cox2b*, or in sequences of *cox2a* amplified from total genomic DNA. Nevertheless, it is still possible that fragments of mtDNA containing the 3-kb inserted region or *cox2* genes have been copied into nuclear genome and became transcribed. An increase in the stability of double-stranded RNAs or the presence of transcription promoter(s) within the insert might also contribute to higher levels of some transcripts. Sequences resembling the 15-bp promoter motif of human mtDNA were found similarly oriented upstream (GCTCCAGAAAAAGGAA) and downstream (TTCAACCAAATTA) of *qnu* and might account, in part, for the increased levels of *qnu* and *orf*3-5 transcripts. Higher levels of *orf*6-9 and *wfw* transcripts might result from the proximity of their corresponding genomic loci to the promoter(s) located within the CR, which, following inversion, were no longer separated from protein-coding genes by a cluster of several tRNA genes that likely slow down the elongation phase of transcription.

### *Qnu* encodes a putative nuclease that might have been actively involved in *cox2* fission

**Fig. 7:**
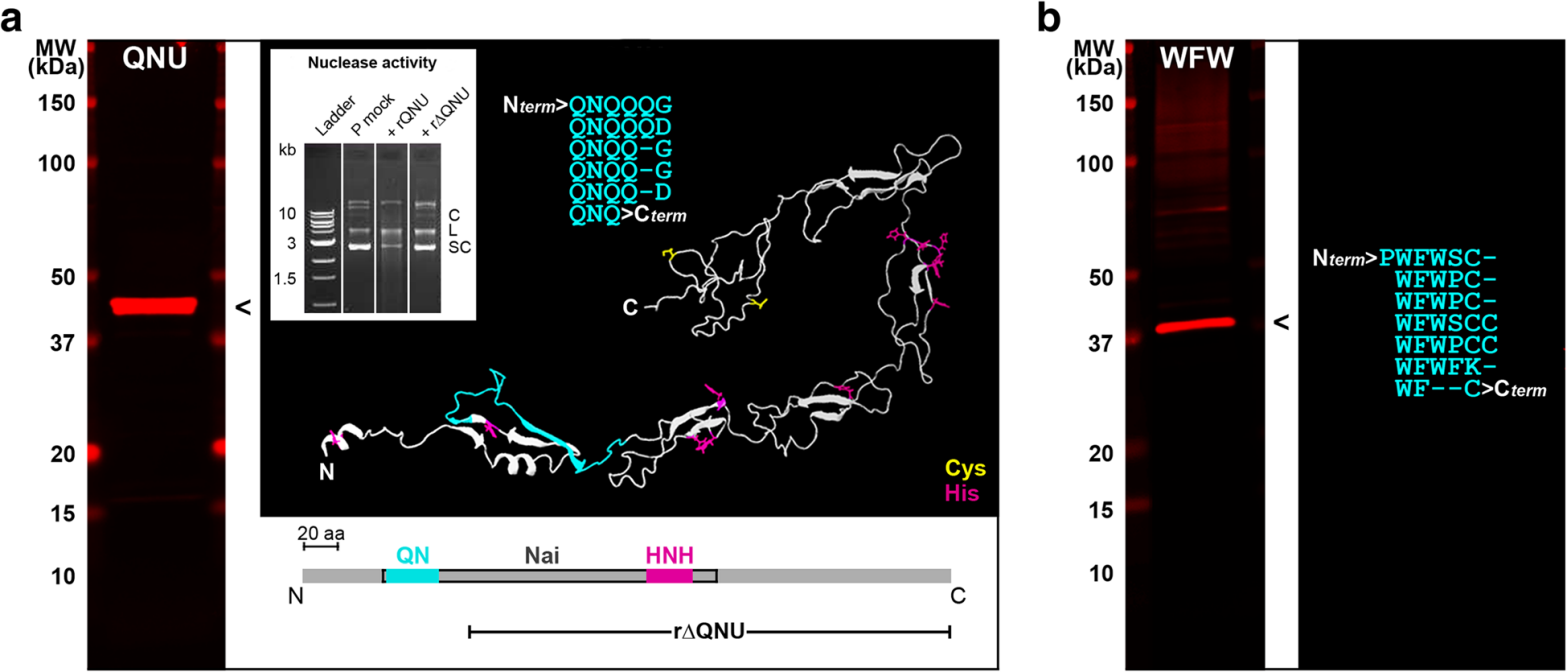
Putative polypeptides encoded by the *cox2*-splitting DNA insert in the *C. p. fossulana* mitogenome. **a** Western blot and ribbon diagram of the I-TASSER-modeled three-dimensional structure of the QNU (the larger of its two isoforms) polypeptide. The tertiary structure was predicted by combining *de novo* and locally applied template-based modeling (PDB templates for local structure predictions were: 1wOrA, 3iymA, 2ocwA, 1pclA, 3cm9S). Signature motif and regions with similarity to nucleic acid-interacting proteins (Nai) and the active site of HNH homing endonucleases (HNH) are indicated on the polypeptide linear model. The inset shows the nuclease activity assay of the recombinant QNU using plasmid DNA as substrate, analyzed by agarose gel electrophoresis. No plasmid degradation was observed in the absence of recombinant proteins (P mock). The addition of rQNU caused a decrease in both SC and C forms of the plasmid and smearing of the L form, indicating at least endonuclease activity of the recombinant QNU (+rQNU). Addition of rΔQNU had no effect on the level of any form of the plasmid, indicating the absence of nuclease activity (+rΔQNU) over a 2-h incubation at 37 °C. Plasmid topology: SC, supercoil; L, linear; C, coil. Deletion of Gln-Asn (QN) repeats suppressed the nuclease activity of the rQNU polypeptide. **b** Western blot of the putative WFW polypeptide and deduced sequence of the repetitive signature motif of WFW that was predicted to adopt helical structure stabilized by Trp residues

Possibility of translation of the inserted ORFs was experimentally addressed for the two largest and best conserved inserted ORFs, *qnu* (Gln-Asn [QN] repeat-containing nuclease gene) and *wfw* (Trp-Phe-Trp [WFW] repeat-encoding putative gene), by western blot (Fig. 7, Additional file 1: Table S3). By this criterion, both ORFs were likely expressed as polypeptides with sizes similar to those predicted from the mapping of their mRNA ends by RACE.

The predicted QNU polypeptide (364 and 387 aa-long isoforms) is hydrophilic (hydropathy value = -0.99) and rich in negatively charged amino acid residues (Fig. 7a). Bioinformatics analysis of its sequence using the BindN server (http://www.web.archive.org/web/20060907042245/bioinformatics.ksu.edu/bindn/) indicated that the Gln-Asn (QN) signature motif-bearing domain and other regions have the potential to interact with DNA and RNA (Additional file 1: Figure S8). In agreement with this prediction, the N-terminal two-thirds of this polypeptide showed sequence similarity to proteins interacting with nucleic acids (Additional file 1: Table S3). A 30-amino acid sequence located within the C-terminal half of the QNU (His^212^-3aa-His-10aa-Asn-9aa-His-3aa-His^241^ in *C. p. fossulana*; KT740996) exhibits features of a nucleolytic domain of homing endonucleases of the HNH family [55]. This domain could potentially form a finger-like structure with a central Asn residue stabilized by a bivalent metal cation coordinating two of its His and/or Cys residues located closer to the C-terminus. Thus, QNU might have been directly involved in *cox2* splitting, functioning as an endonuclease. Pairwise alignment of the sequences around the inserted DNA ends in *C. p. fossulana cox2* revealed the presence of putative remnants of direct repeats (Additional file 1: Figure S9), suggesting that the insertion followed staggered cleavage of the mtDNA, resembling cleavage at a target DNA site generated by homing nucleases.

To further test the possible involvement of QNU in *cox2* fission, its gene was subcloned in an expression vector in *E. coli*, and the purified recombinant QNU polypeptide (rQNU) was assayed for nuclease activity. Two plasmid constructs were prepared, one expressing intact rQNU and the other rΔQNU, without DNA-binding Gln-Asn repeats. In the double-stranded plasmid DNA degradation assay, rQNU, but not rΔQNU, exhibited weak endonuclease activity (Fig. 7a). This result supports, in particular, the role of the QN repeats in interaction of QNU with DNA, although the two recombinant QNU proteins were expressed in *E. coli* and thus differed from the native protein due to differences between genetic codes of invertebrate mitochondria and bacteria.

The other putative polypeptide, WFW (360 aa) (Fig. 7b), has been predicted to be hydrophobic (hydropathy value = 0.15). Interestingly, its deduced amino acid sequence not only exhibits a relatively high number of Cys residues, but they were interspersed with an unusually high number of Trp residues (Fig. 7b). Because of this unusual amino acid composition and lack of sequence similarity to known proteins, the three-dimensional structure and function of WFW cannot currently be predicted reliably, necessitating expression and empirical structural analyses.

## Discussion

**Fig. 8:**
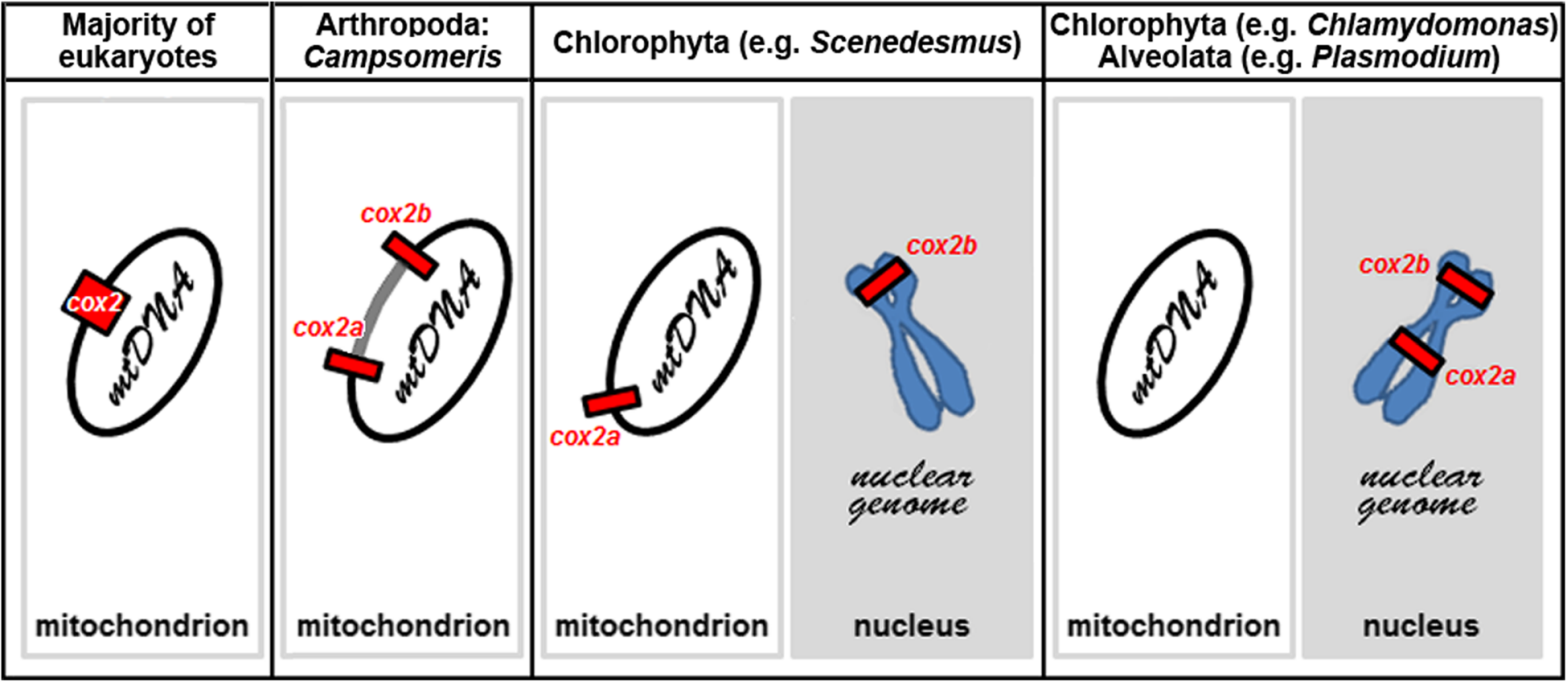
Augmented compilation of the split *cox2* arrangement and its subcellular localization through phylogeny. In the vast majority of eukaryotes, *cox2* is intact and resides in the mtDNA. In wasps *Campsomeris*, *cox2* is split into complementary *cox2a* and *cox2b* genes that reside in the mtDNA. In the chlorophycean algae *Scenedesmus*, *Podohedriella*, *Neochloris*, *cox2* is also split, but *cox2b* had been transferred to the nucleus and lost from the mtDNA. In the chlorophycean algae *Chlamydomonas*, *Polytomella*, *Volvox*, *Haematococcus*, and in apicomplexan parasites, dinoflagellates, and *Perkinsus*, *cox2* is split and both *cox2a* and *cox2b* have been relocated independently of one another to the nuclear genome and lost from the mtDNA

Screening of the fast-evolving mitogenomes of apocritan Hymenoptera for segmental inversions was instrumental in identifying a unique for animals fission of a canonical protein-coding gene, *cox2*, in a genus *Campsomeris* (*Dielis*) of Scoliidae. *Cox2* was split by an equally unique insertion of 3-kb long cluster of multiple ORFs of unknown origin. This evolutionarily recent gene fission, found in the mtDNA of two studied *Campsomeris* species but not in *Scolia* of the same family or in related hymenopteran families, divided *Campsomeris cox2* into two translated genes, *cox2a* and *cox2b*. Such a genomic arrangement has not been found for this gene in the mtDNA of any other organism (Fig. 8). COXIIA and COXIIB polypeptides apparently assemble into a functional COXII heterodimer in a process that may involve interactions in the mitochondrial intermembrane space of COXIIA termini with COXIIB and is likely assisted by other proteins of respiratory complex IV. Although the folding of *Campsomeris* COXIIA and COXIIB has been predicted to be similar to that of *S. bicincta* COXII, COXIIA and, to lesser degree, COXIIB polypeptides exhibit reduced hydrophobicity compared with the corresponding domains of the majority of intact COXII polypeptides. The reduction in hydrophobicity, especially of the first transmembrane helix of COXII, has been shown to be essential for functional import into the mitochondria of COXII encoded in the nucleus [29, 56], but it might also promote intramitochondrial transport of fragmented COXII expressed in the mitochondrial matrix. In particular, Oxa1 is required for the export of the first transmembrane helix of COXII, synthesized in the mitochondrial matrix, to the inner membrane [57]. Similarly, the export of nuclear genome-encoded COXII from the mitochondrial intermembrane space has been proposed to require anchoring of the polypeptide in the inner membrane through its second transmembrane helix and reinsertion of the first helix, which temporarily entered the mitochondrial matrix, depending on Oxa1 [58]. Alternatively, a general decrease in hydrophobicity, especially of COXIIA compared with the N-terminal half of intact COXII, might have evolved to compensate for the original increase in COXIIA hydrophobicity caused by its split from the more hydrophilic C-terminal half of COXII.

The other characteristic of split COXII, namely the increase in Cys content in COXIIB, might facilitate the export of COXIIB to the intermembrane space by inner membrane translocases and chaperones [57] or its interactions with other components of the respiratory complex IV. Moreover, Cys residues might become reversibly oxidized to intra and interpeptide disulfides by, for instance, the intermembrane space MIA pathway [57] to regulate COXII complex assembly and activity in a redox-dependent manner [59, 60].

The 3-kb DNA fragment dividing *Campsomeris cox2* includes several ORFs that are expressed as polyadenylated mRNAs. Four of the ORFs have orthologs in both *Campsomeris* species used in these studies. One of the ORFs, *qnu*, was shown herein to potentially encode a nuclease. The putative polypeptide QNU contains a nucleic acid-binding domain and an HNH-like domain that is present in HNH-class homing endonucleases and may have been directly involved in mediating the split of *cox2*, as the recombinant rQNU exhibited endonucleolytic activity. The presence of remnants of direct repeats flanking the inserted DNA segment further suggested involvement of a homing nuclease in *cox2* fragmentation. Similarly, a homing nuclease encoded by a group I intron located within the *cox1* gene of a basal metazoan, *Metridium* (Cnidaria), was reported to be responsible for genic insertion of the intron [61]. In addition, *in vivo* experiments in yeast showed that endonuclease-encoding introns ensured their own propagation [62]. Examples of non-mitochondrial gene fission caused by insertion of a gene for free-standing homing nuclease mediating fission include split gene of the B-type DNApol of *Methanobacterium* [63] or fragmented *nrdA* gene of *Aeromonas* phage Aeh1 [64]. Alternatively, *Campsomeris cox2* fission might be primarily caused by insertion of other DNA element that provided an integration site for the insertion of 3-kb gene cluster. However, this scenario seems less likely due to the lack of known cases in animals of *cox2* splitting by intervening sequences other than the *Campsomeris* cases reported herein. The implications of the continuing expression of QNU nuclease in the mitochondrial matrix are unknown. The activity of native QNU remains to be determined and might be residual or conditionally induced *in vivo*.

It is currently unclear whether copies of any portion of the *Campsomeris cox2* genes or their 3-kb insert have been transferred to the nuclear genome. To date, no heteroplasmy has been detected for *Campsomeris cox2a*, *cox2b* and new ORFs. However, based on the high levels of some of the transcripts, it cannot be ruled out that the expressed copies, especially of *cox2b* and some inserted ORFs, also reside in the nuclear genome. In some legumes (Angiospermae, Fabaceae), not only do mitochondrial and nuclear copies of *cox2* exist, but in *Dumasia* and a few other genera (mostly Phaseoleae), they are transcribed simultaneously from both genomes [65].

## Conclusion

The discovery of functional fission of *cox2* in the mtDNA of *Campsomeris* highlights the dynamics of mitogenome evolution in Hymenoptera. As a very distinctive character, *cox2* fission can be used to clarify phylogenetic relationships within and among subfamilies of Scoliidae. Importantly, it also raises more general questions concerning the evolution of metazoan mitogenomes and their REDOX systems. Split COXII and the increased number of Cys in COXIIB likely established an additional regulatory mechanism to control OXPHOS by linking COXII assembly and activity to varying levels of reactive oxygen species. Interestingly, the fission of *cox2* occurred through the genic insertion of a relatively large DNA fragment, hence contrary to the general trend of metazoan mitogenome evolution towards a decrease in mtDNA size. The current function, if any, of the ORFs encoded by the *cox2*-splitting insert remains unknown, although four of them have been largely preserved between the two compared *Campsomeris* species. It seems possible that at least QNU, which is encoded by one of these ORFs, might have been involved in *cox2* fission and insert integration into mtDNA, similarly to the role played by mobile element-encoded homing nucleases. Further structural and functional studies of the inserted ORFs might contribute to a better understanding of the mechanisms of insertional mitogenome modifications.

## Methods

### Specimens, isolation of mitochondria, and nucleic acid extraction

The hymenopteran species analyzed herein are listed in Table 1. Voucher specimens were deposited at Texas A&M University (College Station, TX). Intact mitochondria were isolated from thoracic muscles of *C. p. fossulana* using the Qproteome Mitochondria Isolation Kit (Qiagen, Frederick, MD). For DNA preparation, mitochondria or thoracic muscle tissue were lysed in SDS-containing buffer and digested with proteinase K. The lysates were treated with phenol/chloroform, and DNA was precipitated with isopropanol. RNA was extracted using the miRNeasy Mini Kit (Qiagen) and treated with DNaseI (Invitrogen, Carlsbad, CA).

### Screening of mitogenomes for segmental inversions, DNA sequencing, and mtDNA annotation

The PCR primers used to detect inversion were mHCO2198 (5’-TAAAATATAAACTTCAGGGTGWCCAAAAAAYCA-3’), a modification of HCO2198 [66] specific for *cox1*, and HPK16Sbb [67] specific for *rrnL*. The PCR primers used to verify the absence of inversion were mC1-J-1751 (5’-CTCTAATATTGGGAKYACCTGATATAGCWTTCCC-3’), a modification of C1-J-1751 [68] and HPK16Sbb. To minimize the possibility of sequencing NUMTS, circular mitogenomes bearing segmental inversions were first amplified in two overlapping fragments using primers mHCO2198 and HPK16Sbb, and a pair of outward-facing primers complementary to the terminal regions of the fragments amplified with mCO2198 and HPK16Sbb (ouCO2198: 5’-GTAGGAAAAGGAATTGGGACAGGATGAACTA-3’ and ou16S: 5’-GAATAATGACATCCTGAAGATCAGCCAGAA-3’ for *Campsomeris*). Mitogenomes without detected segmental inversion were partially amplified using primers rcCOI-2198 (5’- TTTATTTTGRTTTTTTGGWCACCCTGAAGTTTA-3’) or mC1-J-1751 and HPK16Sbb. PCR was performed using LA Taq DNA polymerase (TaKaRa, Ōtsu, Japan). Reactions were carried out at 94 °C for 30 s and 62 °C for 10 min for 30 cycles. The amplified mtDNA fragments were subsequently used as templates for primer walking. Following direct Sanger sequencing of PCR products, the mitogenomes were assembled using Sequencher v4.8 (Gene Codes, Ann Arbor, MI). Protein- and rRNA-gene boundaries were delimitated by alignment with homologous regions of the mtDNA of other Hymenoptera and, in some cases, by RACE. tRNA genes were identified with tRNAscan-SE 1.21 (http://www.lowelab.ucsc.edu/tRNAscan-SE/). Heteroplasmy was tested by sequencing almost the entire *cox2a* gene amplified from total genomic DNA using the following primers: 5’-TTCAGGATCCAGTATCCCCTAACA-3’ and 5’-AAACCTGAATATTCTGCTGATCAAA-3’, and by analysis of RACE product sequences from *cox2a*, *cox2b*, and inserted ORF transcripts.

### Transcript analyses by RACE and RT-qPCR

The mitochondrial RNA was reverse-transcribed with the SuperScript III First Strand Synthesis System (Invitrogen). The cDNA ends were amplified using SMART-RACE cDNA Amplification Kit (Clontech, Mountain View, CA), cloned into pGEM-T vector (Promega, Madison, WI), and on average 10 clones for each end were sequenced. Primers for qPCR (Additional file 1: Table S4) were designed with PrimerQuest (http://www.idtdna.com/Primerquest/). The *cob* gene was chosen as an internal control. Readings were normalized to *C. p. fossulana cox1* for *Campsomeris* genes or *D. melanogaster cox1* for *Drosophila* genes. Aside from *cob* and *cox1*, only transcripts of similarly oriented genes were converted to cDNAs together using transcript-specific qPCR primers. The qPCR was performed in triplicate using Power SYBR Green PCR Master Mix (Applied Biosystems, Warrington, UK) under the following conditions: incubation at 95 °C for 10 min and 40 cycles of incubation at 95 °C for 15 s and 60 °C for 1 min. For relative quantification, the comparative C_T_ method was used.

### Antibodies and western blot analysis

Polyclonal Abs against synthetic epitopes of the *C. p. fossulana* polypeptides COXIIA, COXIIB, QNU, and WFW were raised in rabbits and affinity-purified (GenScript, Piscataway, NJ). Epitope peptides (COXIIA1: CQWKH{Nle}NFQDPVSPN; COXIIA2: CNGYTYRKLTHGSFI; COXIIB: CSMGVKVDSIPGRLN; QNU: {Nle}NYNHTGQYKTSNC; WFW: CSKP{Nle}FASSSGTG{Nle}NK) were designed using the OptimumAntigen Design Tool (GenScript). Cys residues were added to the N- or C-terminus to facilitate conjugation. Isolated mitochondria (3 μg of protein) were lysed for 10 min at 75 °C in reducing SDS-sample buffer and subjected to 4-20% SDS-PAGE. The proteins were subsequently electrotransferred from the gel to Immobilon FL PVDF membranes (Millipore, Billerica, MA). The western blot signal was detected using primary Abs diluted 1:1000 and Cy5-labeled anti-Rb secondary Abs (Molecular Probes, Eugene, OR) diluted 1:10,000. The membranes were scanned on an Odyssey CLx imager (LI-COR, Lincoln, NE). Antibodies against COXIIA1 and COXIIA2 epitopes recognized the same polypeptide band.

### Subcloning of the *qnu* gene in the bacterial expression vector and QNU activity assay

The full-length *qnu* and its truncated allele, *Δqnu*, missing the N-terminal 30% encoding the DNA-binding QN domain, were PCR amplified using the following primers: qnuR (5’-GAGGTACCTGGATTAATATAATTTTATGGTCGAGGAG-3’), and qnuF1 (5’- AGGGATCCATGAATTATAATCATACTGGTCAATATAAAAC-3’) or qnuF2 (5’- AGGGATCCATGTTACCTCATAATAATAATCTTCCTAATTT-3’), respectively. Upon cleavage with *Kpn*I and *Bam*HI, the amplified products were cloned at the *Kpn*I-*Bam*HI site of the pProEx THb vector (ThermoFisher, Pittsburgh, PA).

The recombinant plasmids were introduced into *E. coli* NiCo21(DE3) (New England Biolabs, Ipswich, MA). Bacteria were grown to the exponential phase, at which point the expression of recombinant proteins was induced with 1 mM IPTG at 30 °C for 6 h. Upon harvesting, the cells were disrupted using xTractor Buffer (Clontech). Recombinant proteins were purified using a CapturemHis-Tagged Purification Kit (Clontech). For the nuclease activity assay, 20 ng of protein was incubated with 400 ng pGEM-derived plasmid in a 20 μl reaction mixture containing 50 mM Tris-HCl (pH 8.0), 150 mM NaCl, and 2 mM MgCl_2_ at 37 °C for 2 h. The samples were then electrophoresed in 1% agarose gel with ethidium bromide and analyzed under UV light.

### Three-dimensional polypeptide structure prediction

Polypeptide tertiary structures were predicted using the I-TASSER algorithm [69] included in the NovaFold software (DNAStar, Madison, WI). The I-TASSER procedure involves multiple threading attempts to match the query and template sequences and *ab initio* folding utilizing the physical characteristics of the query sequence and simulations. Visualization of the polypeptide structures was performed using Lasergen Protean 3D (DNAStar).

## Additional file

Additional file 1: Figure S1. Schematic representation of a PCR-based screen for segmental inversions in mtDNA. **Figure S2.** Alignment of COXII sequences in the region corresponding to the C- and N-termini of *Campsomeris* COXIIA and COXIIB, respectively. **Figure S3.** *C. p. fossulana cox2a* gene. **Figure S4.** *C. p. fossulana cox2b* gene. **Figure S5.** Amino acid residue content in COXII of *C. p. fossulana*, *S. bicincta*, and *A. mellifera*. **Figure S6.** A + T content along the *C. p. fossulana* mtDNA. **Figure S7.** Comparison of QNU and WFW orthologous polypeptides from two *Campsomeris* species. **Figure S8.** *In silico*-determined nucleic acid-binding potential of the *C. p. fossulana* QNU polypeptide. **Figure S9.** Alignment of the *Campsomeris* mtDNA sequences around the *cox2* split site. **Table S1.** Cys residue content of the COXII intermembrane space domain in canonical and modified COXII polypeptides. **Table S2.** Relative synonymous codon usage (RSCU) by mitochondrial genes/ORFs of *C. p. fossulana*. **Table S3.** Amino acid sequence similarities between *C. p. fossulana* polypeptide QNU and nucleic acid-interacting proteins. **Table S4.** Primers used for RT-qPCR. (PDF 1205 kb)

## Abbreviations

aa: amino acid
COX: Cytochrome oxidase
GRAVY: Grand average of hydropathy
mtDNA: mitochondrial genome
NATs: Natural sense antisense transcripts
NUMTS: Nuclear genome-encoded mitochondrial DNA sequences
ORF: Open reading frame
OXPHOS: Oxidative phosphorylation
PDB: Protein data bank
QNU: Gln-Asn repeat-containing nuclease
RACE: Rapid amplification of cDNA ends
WFW: Trp-Phe-Trp repeat-containing putative polypeptide

## References

[1] Gray MW, Burger G, Lang BF (2001). The origin and early evolution of mitochondria. Genome Biol.

[2] Gray MW (2012). Mitochondrial evolution. Cold Spring Harb Perspect Biol.

[3] Wolstenholme DR (1992). Animal mitochondrial DNA: structure and evolution. Int Rev Cytol.

[4] Boore JL (1999). Animal mitochondrial genomes. Nucleic Acids Res.

[5] Saccone C, Gissi C, Reyes A, Larizza A, Sbisà E, Pesole G (2002). Mitochondrial DNA in metazoa: degree of freedom in a frozen event. Gene.

[6] Gissi C, Iannelli F, Pesole G (2008). Evolution of the mitochondrial genome of Metazoa as exemplified by comparison of congeneric species. Heredity.

[7] Bernt M, Braband A, Schierwater B, Stadler PF (2013). Genetic aspects of mitochondrial genome evolution. Mol Phylogenet Evol.

[8] Wey-Fabrizius AR, Podsiadlowski L, Herlyn H, Hankeln T (2013). Platyzoan mitochondrial genomes. Mol Phylogenet Evol.

[9] Ross E, Blair D, Guerrero-Hernández C, Sánchez Alvarado A (2016). Comparative and Transcriptome Analyses Uncover Key Aspects of Coding- and Long Noncoding RNAs in Flatworm Mitochondrial Genomes. G3 (Bethesda).

[10] Breton S, Beaupré HD, Stewart DT, Piontkivska H, Karmakar M, Bogan AE (2009). Comparative mitochondrial genomics of freshwater mussels (Bivalvia: Unionoida) with doubly uniparental inheritance of mtDNA: gender-specific open reading frames (ORFs) and putative origins of replication. Genetics.

[11] Milani L, Ghiselli F, Guerra D, Breton S, Passamonti M (2013). A comparative analysis of mitochondrial ORFans: new clues on their origin and role in species with doubly uniparental inheritance of mitochondria. Genome Biol Evol.

[12] Saccone C, Attimonelli M, Sbisà E (1987). Structural elements highly preserved during the evolution of the D-loop-containing region in vertebrate mitochondrial DNA. J Mol Evol.

[13] Endo K, Noguchi Y, Ueshima R, Jacobs HT (2005). Novel repetitive structures, deviant protein-encoding sequences and unidentified ORFs in the mitochondrial genome of the brachiopod *Lingula anatina*. J Mol Evol.

[14] Wu X, Li X, Li L, Xu X, Xia J, Yu Z (2012). New features of Asian *Crassostrea* oyster mitochondrial genomes: A novel alloacceptor tRNA gene recruitment and two novel ORFs. Gene.

[15] Woischnik M, Moraes CT (2002). Pattern of organization of human mitochondrial pseudogenes in the nuclear genome. Genome Res.

[16] Leister D (2005). Origin, evolution and genetic effects of nuclear insertions of organelle DNA. Trends Genet.

[17] Rogers HH, Griffiths-Jones S (2012). Mitochondrial pseudogenes in the nuclear genomes of *Drosophila*. PLoS One.

[18] Adams KL, Palmer JD (2003). Evolution of mitochondrial gene content: gene loss and transfer to the nucleus. Mol Phylogenet Evol.

[19] Gawryluk RM, Gray MW (2010). An ancient fission of mitochondrial *cox1*. Mol Biol Evol.

[20] Kück U, Jekosch K, Holzamer P (2000). DNA sequence analysis of the complete mitochondrial genome of the green alga *Scenedesmus obliquus*: evidence for UAG being a leucine and UCA being a non-sense codon. Gene.

[21] Nedelcu AM, Lee RW, Lemieux C, Gray MW, Burger G (2000). The complete mitochondrial DNA sequence of *Scenedesmus obliquus* reflects an intermediate stage in the evolution of the green algal mitochondrial genome. Genome Res.

[22] Pérez-Martínez X, Antaramian A, Vazquez-Acevedo M, Funes S, Tolkunova E, d’Alayer J (2001). Subunit II of cytochrome c oxidase in Chlamydomonad algae is a heterodimer encoded by two independent nuclear genes. J Biol Chem.

[23] Funes S, Davidson E, Reyes-Prieto A, Magallón S, Herion P, King MP, González-Halphen D (2002). A green algal apicoplast ancestor. Science.

[24] Rodríguez-Salinas E, Riveros-Rosas H, Li Z, Fucíková K, Brand JJ, Lewis LA, González-Halphen D (2012). Lineage-specific fragmentation and nuclear relocation of the mitochondrial *cox2* gene in chlorophycean green algae (Chlorophyta). Mol Phylogenet Evol.

[25] Adams KL, Ong HC, Palmer JD (2001). Mitochondrial gene transfer in pieces: fission of the ribosomal protein gene *rpl2* and partial or complete gene transfer to the nucleus. Mol Biol Evol.

[26] Gawryluk RM, Gray MW (2009). A split and rearranged nuclear gene encoding the iron-sulfur subunit of mitochondrial succinate dehydrogenase in Euglenozoa. BMC Res Notes.

[27] Claros MG, Perea J, Shu Y, Samatey FA, Popot JL, Jacq C (1995). Limitations to *in vivo* import of hydrophobic proteins into yeast mitochondria. The case of a cytoplasmically synthesized apocytochrome b. Eur J Biochem.

[28] Herrmann JM, Koll H, Cook RA, Neupert W, Stuart RA (1995). Topogenesis of cytochrome oxidase subunit II. Mechanisms of protein export from the mitochondrial matrix. J Biol Chem.

[29] Daley DO, Clifton R, Whelan J (2002). Intracellular gene transfer: reduced hydrophobicity facilitates gene transfer for subunit 2 of cytochrome c oxidase. Proc Natl Acad Sci U S A.

[30] Burger G, Zhu Y, Littlejohn TG, Greenwood SJ, Schnare MN, Lang BF, Gray MW (2000). Complete sequence of the mitochondrial genome of *Tetrahymena pyriformis* and comparison with *Paramecium aurelia* mitochondrial DNA. J Mol Biol.

[31] Edqvist J, Burger G, Gray MW (2000). Expression of mitochondrial protein-coding genes in *Tetrahymena pyriformis*. J Mol Biol.

[32] Swart EC, Nowacki M, Shum J, Stiles H, Higgins BP, Doak TG, Schotanus K (2011). The *Oxytricha trifallax* mitochondrial genome. Genome Biol Evol.

[33] Rayapuram N, Hagenmuller J, Grienenberger JM, Bonnard G, Giegé P (2008). The three mitochondrial encoded CcmF proteins form a complex that interacts with CCMH and c-type apocytochromes in *Arabidopsis*. J Biol Chem.

[34] Dowton M, Austin AD (1999). Evolutionary dynamics of a mitochondrial rearrangement “hot spot” in the Hymenoptera. Mol Biol Evol.

[35] Xiao JH, Jia JG, Murphy RW, Huang DW (2011). Rapid evolution of the mitochondrial genome in Chalcidoid wasps (Hymenoptera: Chalcidoidea) driven by parasitic lifestyles. PLoS One.

[36] Kaltenpoth M, Showers Corneli P, Dunn DM, Weiss RB, Strohm E, Seger J (2012). Accelerated evolution of mitochondrial but not nuclear genomes of Hymenoptera: new evidence from crabronid wasps. PLoS One.

[37] Davila JI, Arrieta-Montiel MP, Wamboldt Y, Cao J, Hagmann J, Shedge V (2011). Double-strand break repair processes drive evolution of the mitochondrial genome in Arabidopsis. BMC Biol.

[38] Cameron SL (2014). Insect mitochondrial genomics: implications for evolution and phylogeny. Annu Rev Entomol.

[39] Oudot-Le Secq MP, Fontaine JM, Rousvoal S, Kloareg B, Loiseaux-De GS (2001). The complete sequence of a brown algal mitochondrial genome, the ectocarpale *Pylaiella littoralis* (L.) Kjellm. J Mol Evol.

[40] Waller RF, Keeling PJ, van Dooren GG, McFadden GI (2003). Comment on “A green algal apicoplast ancestor”. Science.

[41] Vallès Y, Halanych KM, Boore JL (2008). Group II introns break new boundaries: presence in a bilaterian’s genome. PLoS One.

[42] Hackett JD, Yoon HS, Soares MB, Bonaldo MF, Casavant TL, Scheetz TE (2004). Migration of the plastid genome to the nucleus in a peridinin dinoflagellate. Curr Biol.

[43] Waller RF, Keeling PJ (2006). Alveolate and chlorophycean mitochondrial *cox2* genes split twice independently. Gene.

[44] Shiba K, Schimmel P (1992). Functional assembly of a randomly cleaved protein. Proc Natl Acad Sci U S A.

[45] Popot JL, Engelman DM (2000). Helical membrane protein folding, stability, and evolution. Annu Rev Biochem.

[46] Mackenzie KR (2006). Folding and stability of alpha-helical integral membrane proteins. Chem Rev.

[47] Iwata S, Ostermeier C, Ludwig B, Michel H (1995). Structure at 2.8 A resolution of cytochrome c oxidase from *Paracoccus denitrificans*. Nature.

[48] Magliery TJ, Wilson CG, Pan W, Mishler D, Ghosh I, Hamilton AD, Regan L (2005). Detecting protein-protein interactions with a green fluorescent protein fragment reassembly trap: scope and mechanism. J Am Chem Soc.

[49] Schmidt TR, Wildman DE, Uddin M, Opazo JC, Goodman M, Grossman LI (2005). Rapid electrostatic evolution at the binding site for cytochrome c on cytochrome c oxidase in anthropoid primates. Proc Natl Acad Sci U S A.

[50] Katayama S, Tomaru Y, Kasukawa T, Waki K, Nakanishi M, Nakamura M (2005). Antisense transcription in the mammalian transcriptome. Science.

[51] Soldà G, Suyama M, Pelucchi P, Boi S, Guffanti A, Rizzi E (2008). Non-random retention of protein-coding overlapping genes in Metazoa. BMC Genomics.

[52] Faure E, Delaye L, Tribolo S, Levasseur A, Seligmann H, Barthélémy RM (2011). Probable presence of an ubiquitous cryptic mitochondrial gene on the antisense strand of the cytochrome oxidase I gene. Biol Direct.

[53] Mercer TR, Neph S, Dinger ME, Crawford J, Smith MA, Shearwood AM (2011). The human mitochondrial transcriptome. Cell.

[54] Zhan S, Lukens L (2013). Protein-coding cis-natural antisense transcripts have high and broad expression in Arabidopsis. Plant Physiol.

[55] Mehta P, Katta K, Krishnaswamy S (2004). HNH family subclassification leads to identification of commonality in the His-Me endonuclease superfamily. Protein Sci.

[56] Supekova L, Supek F, Greer JE, Schultz PG (2010). A single mutation in the first transmembrane domain of yeast COX2 enables its allotopic expression. Proc Natl Acad Sci U S A.

[57] Fox TD (2012). Mitochondrial protein synthesis, import, and assembly. Genetics.

[58] Jiménez-Suárez A, Vázquez-Acevedo M, Rojas-Hernández A, Funes S, Uribe-Carvajal S, González-Halphen D (2012). In *Polytomella* sp. mitochondria, biogenesis of the heterodimeric COX2 subunit of cytochrome c oxidase requires two different import pathways. Biochim Biophys Acta.

[59] Biswas S, Chida AS, Rahman I (2006). Redox modifications of protein-thiols: emerging roles in cell signaling. Biochem Pharmacol.

[60] Koehler CM, Tienson HL (2009). Redox regulation of protein folding in the mitochondrial intermembrane space. Biochim Biophys Acta.

[61] Beagley CT, Okimoto R, Wolstenholme DR (1988). The mitochondrial genome of the sea anemone *Metridium senile* (Cnidaria): introns, a paucity of tRNA genes, and a near-standard genetic code. Genetics.

[62] Colleaux L, D’Auriol L, Galibert F, Dujon B (1988). Recognition and cleavage site of the intron-encoded omega transposase. Proc Natl Acad Sci U S A.

[63] Kelman Z, Pietrokovski S, Hurwitz J (1999). Isolation and characterization of a split B-type DNA polymerase from the archaeon *Methanobacterium thermoautotrophicum* deltaH. J Biol Chem.

[64] Friedrich NC, Torrents E, Gibb EA, Sahlin M, Sjöberg BM, Edgell DR (2007). Insertion of a homing endonuclease creates a genes-in-pieces ribonucleotide reductase that retains function. Proc Natl Acad Sci U S A.

[65] Adams KL, Song K, Roessler PG, Nugent JM, Doyle JL, Doyle JJ, Palmer JD (1999). Intracellular gene transfer in action: dual transcription and multiple silencings of nuclear and mitochondrial *cox2* genes in legumes. Proc Natl Acad Sci U S A.

[66] Folmer O, Black M, Hoeh W, Lutz R, Vrijenhoek R (1994). DNA primers for amplification of mitochondrial cytochrome c oxidase subunit I from diverse metazoan invertebrates. Mol Mar Biol Biotechnol.

[67] Hwang UW, Park CJ, Yong TS, Kim W (2001). One-step PCR amplification of complete arthropod mitochondrial genomes. Mol Phylogenet Evol.

[68] Simon C, Frati F, Beckenbach A, Crespi B, Liu H, Flook P (1994). Evolution, weighting, and phylogenetic utility of mitochondrial gene sequences and a compilation of conserved polymerase chain reaction primers. Ann Entomol Soc Am.

[69] Zhang Y (2008). I-TASSER server for protein 3D structure prediction. BMC Bioinformatics.

[70] He D, Fiz-Palacios O, Fu CJ, Fehling J, Tsai CC, Baldauf SL (2014). An alternative root for the Eukaryote tree of life. Curr Biol.

[71] Mao M, Gibson T, Dowton M (2015). Higher-level phylogeny of the Hymenoptera inferred from mitochondrial genomes. Mol Phylogenet Evol.

[72] Johnson BR, Borowiec ML, Chiu JC, Lee EK, Atallah J, Ward PS (2013). Phylogenomics resolves evolutionary relationships among ants, bees, and wasps. Curr Biol.

[73] Sonnhammer ELL, von Heijne G, Krogh A. A hidden Markov model for predicting transmembrane helices in protein sequences. In: Glasgow J, et al., editors. Proc. Sixth Int. Conf. Intelli. Syst. Mol. Biol. Menlo Park (CA): AAAI Press; 1998. p. 175-82. 9783223

