# Supplementary material for: Evolutionarily recent, insertional fission of mitochondrial cox2 into complementary genes in bilaterian Metazoa
Source: BMC Genomics. 2017 Mar 31;18:269. doi: 10.1186/s12864-017-3626-5 (PMC5374615; doi:10.1186/s12864-017-3626-5)
Supplement: Additional file 1: Figure S1. — Schematic representation of a PCR-based screen for segmental inversions in mtDNA. Figure S2. Alignment of COXII sequences in the region corresponding to the C- and N-termini of Campsomeris COXIIA and COXIIB, respectively. Figure S3. C. p. fossulana cox2a gene. Figure S4. C. p. fossulana cox2b gene. Figure S5. Amino acid residue content in COXII of C. p. fossulana, S. bicincta, and A. mellifera. Figure S6. A + T content along the C. p. fossulana mtDNA. Figure S7. Comparison of QNU and WFW orthologous polypeptides from two Campsomeris species. Figure S8. In silico-determined nucleic acid-binding potential of the C. p. fossulana QNU polypeptide. Figure S9. Alignment of the Campsomeris mtDNA sequences around the cox2 split site. Table S1. Cys residue content of the COXII intermembrane space domain in canonical and modified COXII polypeptides. Table S2. Relative synonymous codon usage (RSCU) by mitochondrial genes/ORFs of C. p. fossulana. Table S3. Amino acid sequence similarities between C. p. fossulana polypeptide QNU and nucleic acid-interacting proteins. Table S4. Primers used for RT-qPCR. (PDF 1205 kb) [file 12864_2017_3626_MOESM1_ESM.pdf]

Additional files

Evolutionary recent insertional split of the mitochondrial *cox2* into complementary genes in bilaterian Metazoa

Przemyslaw Szafranski

Department of Molecular and Human Genetics, Baylor College of Medicine, Houston, Texas 77030, USA

Supplementary figures 1-10

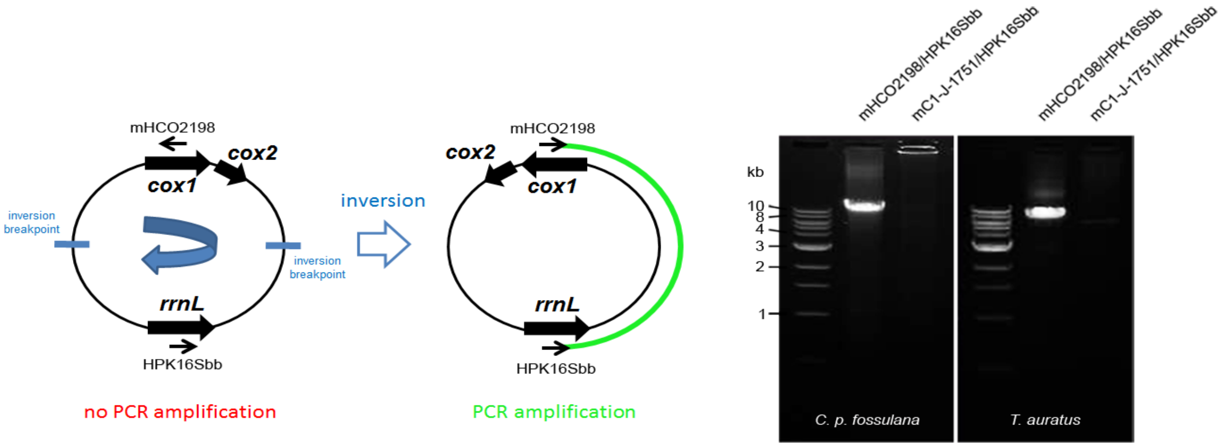

Figure S1. Schematic representation of a PCR-based screen for segmental inversions in mtDNA. Primer HPK16Sbb was used in all PCR reactions. The second primer was either mHCO2198, which allowed for amplification of mtDNA only if there was an inversion of *cox1* versus *rrnL*, or its reverse complement, rcCOI-2198, or mC1-J-1751, which allowed for amplification in the absence of the inversion. An example of screen PCR reactions, analyzed by 1% agarose gel electrophoresis, shows amplification of 12 and 9 kb fragments from mtDNA of *C. p. fossulana* and *T. auratus*, respectively, using primers mHCO2198 and HPK16Sbb that indicates the presence of inversions of *cox1* versus *rrnL*.

```
Hymenoptera:                  Campsomeris p. fossulana     COXIIA      TVKVMGNQWFWSAEYSGFD----------------------------------
                              Campsomeris p. fossulana     COXIIB      ---------------------------------------------MCFRLLDT
                              Campsomeris sp. HA10513      COXIIA[a]   TVKVLGNQWFWTAEYSGFDSNSMDFD------MY
                              Campsomeris sp. HA10513      COXIIB[a]                                       MKYLKKKGKKLFRLLDT
                              Scolia bicincta                          TIKMIGNQWFWIAEYQDMIIN-KSFNNPLSIEMYMSSDINK----GMFRLLET
                              Scolia dubia                             TIKMIGNQWFWVAEYQDMIIN-KSFNNPLSIEMYMSSDINK----GMFRLLET
                Aculeata      Wallacidia oculata                       TIKSVGHQWYWTYEYPDFMK---TFD---SYMMTEKSQKK-------FRLMDV
                              Myzinum maculatum                        TLKSTGHQWYWTFEYSDIYC--MEFD---SFMIPSQD-NQ-------FRLLDV
                              Solenopsis geminata                      SIKSIGHQWYWSYEYSDFPS--IEFD---SFMIPSDQLMA-----NEFRLLDV
                              Apis cerana                              SVKSIGHQWYWSYEYPEFNN--IEFD---SYMLNYSNLNQ-------FRLLET
                              Bombus ignitus                           SIKSIGHQWYWSYEYPEFNN--YEFD---SYMLNYKSMNQ-------FRLLET
                              Polistes sp.                             TIKSIGHQWYWSYEYSDYKN--INFD---SYMINFNKNNLSQ-----FRLLDV
                              Chrysis sp.                              TIKAVGHQWYWSYEYSDLKI--LDFD---SYMLDEDTVNLN----NFFRLLDV
                              Cephalonomia gallicola                   TIKTIGHQWYWSYEYSDFNN--LNFD---SFMLNKMNKNN-----YFMRLLDV
                              Schletterius cinctipes                   TIKTIGNQWYWNYEYSDFKN--IEFS---SFMIKNYKKTE------SFRLLDV
                              Evania appendigaster                     NVKISGHQWYWSYEIKEFN---IEFD---SFLNSSLSP-------GSFRLLDT
                              Cotesia vestalis                         TIKILGHQWYWSYEFSDFFM--VEFD---SFMLKDYMMN-------NFRLLDV
                              Spathius agrili                          TLKIIGHQWYWSYEYSDFKN--VEFD---SFMISEFENKN------LFRLLDV
                "Parasitica"  Macrocentrus camphoraphilus              MIKILGHQWYWSYEYSDFLE--MSFD---SFMIKNFNLD-------NFRLLDV
                              Aphidius gifuensis                       TLNILGHQWYWSYEYVDFKN--LSFD---SFMIQDNFIDL-----GNYRLLEV
                              Diadegma semiclausum                     TIKSIGHQWYWSYEYSDFKN--INFN---SFMIKNFNKHN-------FRLLDV
                              Enicospilus sp.                          TIKSIGHQWYWSYEYPDFNN--INFN---SFMIKNFNLNN-------FRLLEV
                              Vanhornia eucnemidarum                   TIKSIGHQWYWSYEYPEFNN--FSFD---SFMLKDNSSTN------NFRLLDT
                              Nasonia vitripennis                      TIKIIGHQWYWSYEYSDFKM--INFD---SFMIKNNNM---------FRLLDV
                Symphyta      Philotrypesis pilosa                     SIKVMGHQWYWSYELNDFKS--INFD---SFMIKDENNNDL------FRLLDV
                              Orussus occidentalis                     SIKIIGNQWYWKYEYNDISNKSIEIE---SFMEPESSSIKN-----SFRLIDV
                              Cephus cinctus                           TIKCMAHQWYWSYEYSDFNN--IEFD---SFMIPTEELNKN-----EFRLLEV
Diptera:                      Drosophila melanogaster                  TLKSIGHQWYWSYEYSDFNN--IEFD---SYMIPTNELMTD-----GFRLLDV
Psocodea:                     Lepidopsocid sp.                         TLKTIGHQWYWSYEYSDFNN--IEFD---SFMIPSNENFNS-----DFRLLEV
"Phthiraptera":               Pediculus humanus[b]                     SLKVVGHQWFWSYEYGDWEN--IEFD---SYMMKLEELDSS----CPFRLLEA
Thysanoptera:                 Thrips imaginis                          SVKVFGAQWYWIYENFDMTKG-NSYS---SYMIPDNELLKGKPCNMGWRLLQT
Hemiptera:                    Neomaskellia andropogonis                TCKVIGHQWFWSYELSDFMD--LGFD---SYMIIN-----------LARFLEV
Dermaptera:                   Challia fletcheri                        TLKVVGHQWYWSYEYSDFKE--VEFD---SYMIMSNDLEEG-----YFRLLDV
Blattodea:                    Periplaneta fuliginosa                   TLKTIGHQWYWSYEYSDFAK--VEFD---SYMIPQNEMENN-----MFRLLDV
Archaeognatha:                Petrobius brevistylis                    TLKTIGHQWYWSYEYSDFMN--VEFD---SYMIQTKNLENN-----GFRLLDV
Diplura:                      Lepidocampa weberi                       TIKAIGHQWYWSYEYSDFNS--IEFD---SYMIPTPDLELN-----GFRLLEV
Chlorophyta:                  Chlamydomonas reinhardtii    COXIIA      TVKIIGRQWYWSYEMHDH>C-terminal extension
                              Chlamydomonas reinhardtii    COXIIB                 N-terminal extension<FDSYMLT--EVQPGQLRVLEV
                              Trypanosoma gondii           COXIIA      TVKVIGRQWYWIYEVESP>C-terminal extension
Alveolata:                    Trypanosoma gondii           COXIIB                 N-terminal extension<FQSNMVTDEDLLPGMLRNLEV
                              Tetrahymena pyriformis       COXII       TVRVRARQWYWIYKFELKN>   insertion     <GELIPVTLARRILRT

                                                                       *** ***********  **      **   ****              ******
```

[a] The ends of *C.* sp. HA10513 *cox2a* and *cox2b* were deduced based on sequence comparison with *cox2* of other species.

[b] The mtDNA of *P. humanus* consists of 18 minicircular chromosomes. Minichromosome bearing the *cox2* gene is 0.8 kb long and contains yet only the *trnY* gene.

Figure S2. Alignment of COXII sequences in the region corresponding to the C- and N-termini of *Campsomeris* COXIIA and COXIIB, respectively. Asterisks denote high level of amino acid residue conservation. Positions of the most conserved residues are highlighted in red. Dashes indicate gaps inserted to improve alignment. The COXII split occurred within its less conserved region that apparently tolerates such disruption.

cox1>AAGAAATTCCTGTAATTTTTAAA**TAAA**caaa**taa**tagtttattaattaaacat**taa**atttt**taa**ttt

E I P V I F K *

cox2a

aataatgctttgttatcttaTGAAGCTATTTGTG**ATG**TTTCAATGAAAAATAATAAATTTTCAGGATCCAG

M F Q W K M M N F Q D P V

TATCCCCTAACATACAAGCTATAATTGGATTTCATGATTTAATTATATGCATTACTATTATAATCATCATT

S P N M Q A M I G F H D L I M C I T I M I I I

TTAATTGTTTATAACTATTATTTTATCTCAAATAATGGATATACATACCGAAAATTAACTCACGGTAGATT

L I V Y N Y Y F I S N N G Y T Y R K L T H G S F

TATCGAAGCAATTTGAACAATACTACCTATTATTATCCTTGTACTTTTATCGATTCCTTCAATAAAAATTT

I E A I W T M L P I I I L V L L S I P S M K I L

TATACATAAATGATGAAGGTACATTAAATCCTAGTATAACTGTTAAAGTCATAGGAAATCAATGATTTTGA

Y M N D E G T L N P S M T V K V M G N Q W F W

TCAGCAGAATATTCAGGTTTTGAT**T**CTAATTCTATAGAATTTGATTTATTTTTAAATAAAGCACTTAAAGA

S A E Y S G F D *

Figure S3. *C. p. fossulana cox2a* gene. Bases corresponding to RACE-determined polyadenylated nucleotides highlighted in red. A likely remnant of the *trnL*^{TTA} gene is shown in lower case letters. The *cox1* gene has backup stop codons in all three ribosome reading frames.

```
                            cox2b
TATTAAATATTTTAAAAAAAAAAGTAAAATATGTTTTCGATTATTAGATACTGATCGTCGAATAGTTGTACCAATCAATAACCCTATTCGTATTTTAACTTCTTCATTAGATGTAATT
                            M  C  F  R  L  L  D  T  D  R  R  M  V  V  P  I  N  N  P  I  R  I  L  T  S  S  L  D  V  I
CATTCTTTTGCAATTCCTTCAATAGGAGTAAAGGTAGACAGAATTCCTGGTCGATTAAATCAAAGTTTCTTATATTGCCAACAAATAGGAGTATTTTTTGGACAATGTTCAGAAATTTG
H  S  F  A  I  P  S  M  G  V  K  V  D  S  I  P  G  R  L  N  Q  S  F  L  Y  C  Q  Q  M  G  V  F  F  G  Q  C  S  E  I  C
TGGATTAAATCATAGATATATACCTTTTTGTATTGAAGTCACTAATTATGAAAACTTCTTAGAATGATTTAAAAAAGTTGGAAAAAAAATATTAATTTTTCAACTATACAATTTATAATT
 G  L  N  H  S  Y  M  P  F  C  I  E  V  T  N  Y  E  N  F  L  E  W  F  K  K  V  G  K  K  Y
TAAACTTTTTATTTATCCTCAATTCTTCAACGGGAGGTAAGAGGTATCCTCCCCTTAGCCTTATAGGCTAAGCATTTTGTAACTAATATTTAAAGTATAGGCCCTTAGCCTACAATAGT
AAGTTAAAAGTCTTACTCAAAATGTCTTAATTAAATAAATTATTCAAAATGAGTAATAAATCTTTCAATTCTTAATTTACTTCCAATCAAAATTAAATTATTGATCTTCTTATATTAC
```

Figure S4. *C. p. fossulana cox2b* gene. RACE-determined polyadenylated mRNA termini correspond to nucleotides highlighted in red. The sequence TAAG adjacent to the *trnK*^{AAG} gene (shaded gray), particularly its guanine residue, corresponds to the most frequently polyadenylated *cox2b* mRNA 3' end. The *trnM*^{ATA} gene (coding for tRNA missing the TψC (T) arm) that overlaps with the 3' end of *cox2b* and is encoded on the same strand as *cox2b* is underlined. The *trnM*^{ATA} gene almost perfectly overlaps with antiparallel-encoded *trnF*^{TCC} (not shown).

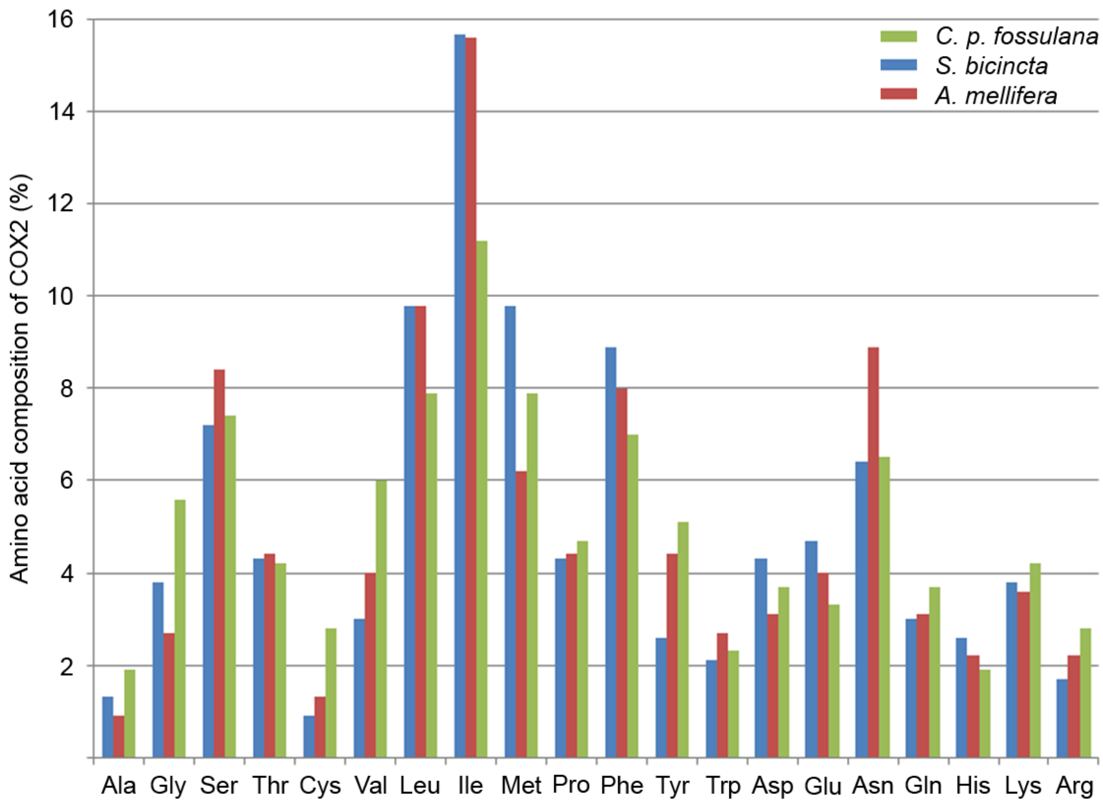

Figure S5. Amino acid residue content in COXII of *C. p. fossulana*, *S. bicincta*, and *A. mellifera*.

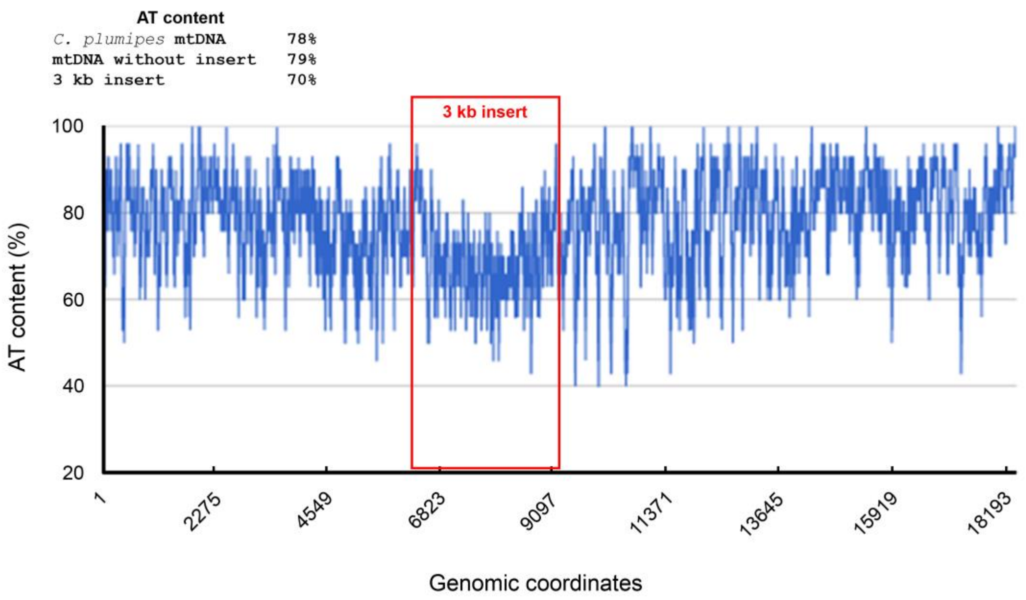

Figure S6. A+T content along the *C. p. fossulana* mtDNA. Genomic coordinates refer to the strand that encoded the majority of protein genes (KT740996). The mitogenome was arbitrary linearized as in Fig. 2. The A+T distribution was determined with GC content calculator (http://www.biologicscorp.com/tools/GCContent/) over a 30 base window. The A+T content is reduced by ~13% within the 3 kb insert (red frame).

QNU

```
C. p. fossulana   3 YNHTGQYKTSNLFI-PVP---DEDA-NMGLLLDDIDNEIDDEGGGDAVMQNHLNQNQQQGQNQQQ--
                        GQ     L   PVP   D    N   + D+ D E DDEG    VMQNHL QN    QN
C. sp. HA10513    1 MSCSGQHGMDGLIDAPVPNNVDFNIHNEDDIDDESDDEDDDEG---VVMQNHLDQNHL-DQNNLDQN

C. p. fossulana  63 --------------------------------DQNQQGQNQQGQNQQDQNQ-GIPNPNNQLPPPHLN
                                                    DQN   QN   QN  DQNQ GIPNPNN LP PHLN
C. sp. HA10513   59 NLDQNNLDQNNLDQNNLDQNNLDQNNLDQNNLDQNNLDQNNLDQNNLDQNQQGIPNPNNRLPLPHLN

C. p. fossulana  97 PNNPL----PHNNNL-PNLQPFNPEMLPNVPQQVPQGNGHAFPDVPFDQQIDPQMEPPMIIIEDPEQ
                    PNNPL    PHNNN+ PNL PFNPEMLPNVPQQVPQGNGHAFPDVPFDQQ+DPQMEPPMIIIEDPE+
C. sp. HA10513  131 PNNPLPPQVPHNNNIPPNLLPFNPEMLPNVPQQVPQGNGHAFPDVPFDQQVDPQMEPPMIIIEDPEE

C. p. fossulana 159 FQNGFPLFDLNNPEGENQIPLLQPLIDPDNQDQNNHFPPIVNGDQQDQPMENNHHNHHNFELPDFDD
                    FQNGFP+FD NNPEGENQIPLLQPLIDPDNQDQNNHFPPIVNGDQQ +  E   H+HH+FELPD +D
C. sp. HA10513  198 FQNGFPMFDPNNPEGENQIPLLQPLIDPDNQDQNNHFPPIVNGDQQMEEEEEDHHHHH-FELPDIED

C. p. fossulana 226 DNAQAGPLVNGHVPEHEQLLILPPFNIDPIPPDESSSSISSSIRMSDSSGSMADDEEEYSLPDNRFL
                    DNAQ  PLVNGHVPEHEQLL+LPPFN +PIPPDE SSS+SS   MSD  GS ADDEEE +    RF+
C. sp. HA10513  264 DNAQE-PLVNGHVPEHEQLLVLPPFNNNPIPPDEESSSSNSSHHMSDGEGSFADDEEELAPMWWRFM
```

WFW

```
C. p. fossulana  88 MSNRLSGKLYSSSSSAMDPDLSLMRILLLILLLLSS-GGIGSMLNGGKINSCSCSGTWPLTKGPAWA
                    M NR      SSSSSA DP  SLM  LL  LLLLSS GGIG +LNGGK N+CSCSGTWPLTKG +WA
C. sp. HA10513    1 M-NRHHMGANSSSSSAKDPSPSLMWCLLF-LLLLSSSGGIGLLLNGGKTNNCSCSGTWPLTKG-SWA

C. p. fossulana 154 LSSSKSGNSKLWWLWWLFSMGWSCWSPFTMGGKWLFWSWLSGSIKGWSKGIWFSPSGLFSSNNGNPF
                    LSSS SGNSK WW WW  S    CWSPFTMGGKWLFWSWLSGSI GWSKGIWFSPSGLF SN GNPF
C. sp. HA10513   65 LSSSMSGNSKWWW-WWSSSSSSICWSPFTMGGKWLFWSWLSGSINGWSKGIWFSPSGLFGSNIGNPF

C. p. fossulana 221 WNCSGSSMMIMGGSIWGSICWSKGTSGKAWPFPWGTCWGTLGSISGLNGCKLGSLLLW-----GKGL
                    WN SGSSMMIMGGSIWGS CWSKGTSGKAWPFPWGTCWGTLGSISGLNG KLG +LL      G GL
C. sp. HA10513  131 WNSSGSSMMIMGGSIWGSTCWSKGTSGKAWPFPWGTCWGTLGSISGLNGSKLGGMLLLWGTCGGNGL

C. p. fossulana 283 LGFKWGGGNWLFGLGMP----------------WFWSCWFWPCWFWPCWFWSCCW----FWPCC---
                    LGF WG GN LFGLGMP                 FWS  FW   FW   FWS  +    FW
C. sp. HA10513  198 LGFSWGSGNRLFGLGMPCWFWSKLFWSKLFWSKLFWSKLFWSKLFWSKLFWSKLFWSKLFWSKLFWS

C. p. fossulana 327 -----------WFWFKWFCITASPPPSSSISLS--MSSSSKPMFASSS-GTGMNK-LLVLYWPVWL
                               WFW KWFCIT  P  SSS SLS  MSSS   M  S   GTG      +  WP  L
C. sp. HA10513  265 KLFWSKLFWSKWFWSKWFCIT-TPSSSSSSSLSSSMSSSLW-MLKSTLLGTGASINPSIPCWPEQL
```

Figure S7. Comparison of QNU and WFW orthologous polypeptides from two *Campsomeris* species. *C. p. fossulana* QNU: 364/387 aa, WFW: 377 aa; *C.* sp. HA10513 QNU: 333 aa, WFW: 359aa. Gln-Asn repeats of QNU and Trp-containing repeats of WFW are highlighted in blue, key residues of the HNH site of QNU are highlighted in purple.

DNA-binding

```
Sequence:       MNYNHTGQYKTSNLFIPVPDEDANMGLLLDDIDNEIDDEGGGDAVMQNHLNQNQQQGQNQ
Prediction:     -++++++++++++---------------------------------+-+-++++++-+++
Confidence:     527437378876558999999999799999999999999999988887252723343444433

Sequence:       QQDQNQQGQNQQGQNQQDQNQGIPNPNNQLPPPHLNPNNPLPHNNNLPNLQPFNPEMLPN
Prediction:     ++-++++-++++-++++-+-+---+-+++--+-+-++++---++----+-+---------
Confidence:     335332354435432335242674243236425272222554224475282565667864

Sequence:       VPQQVPQGNGHAFPDVPFDQQIDPQMEPPMIIIEDPEQFQNGFPLFDLNNPEGENQIPLL
Prediction:     ---+--+-+-+------------------------------+------------------
Confidence:     954275363828689989985998599799999999996762888888965768766977 9

Sequence:       QPLIDPDNQDQNNHFPPIVNGDQQDQPMENNHHNHHNFELPDFDDDNAQAGPLVNGHVPE
Prediction:     ------------------------------+------------------------+-----
Confidence:     699999975644458779968865755674244456689999999969587478285978

Sequence:       HEQLLILPPFNIDPIPPDESSSSISSSIRMSDSSGSMADDEEEYSLPDNRFLMERQSGRR
Prediction:     -------------------+++++++++++++++++-------------+---++++-++
Confidence:     899999987979879844445772976283734423799999954757747872735489

Sequence:       PMFNQIIGDLSDNEMMVDQVDEDNENFVQEMLDADCLPVEVEIQVVPNESVNIPLCPPFP
Prediction:     ++--------+---------------------------------------+---------
Confidence:     327659988927689999999998988979999999998999998985473948577677 7

Sequence:       DFMNSDSDEEYVPRPLPTPIFNLTPRP
Prediction:     ----+-----+--++--+---+-++++
Confidence:     868426475828672553464368293
```

RNA-binding

```
Sequence:       MNYNHTGQYKTSNLFIPVPDEDANMGLLLDDIDNEIDDEGGGDAVMQNHLNQNQQQGQNQ
Prediction:     -++++++++++++----------+------------------+-----+-+++++++++++
Confidence:     733357686765485859786662889998999689878656266555364545785877

Sequence:       QQDQNQQGQNQQGQNQQDQNQGIPNPNNQLPPPHLNPNNPLPHNNNLPNLQPFNPEMLPN
Prediction:     +++++++++++++++++++++++++++++-++++-+++++++++++-++-+--+------
Confidence:     775656758778586673543225434445332454223323233523835627889555

Sequence:       VPQQVPQGNGHAFPDVPFDQQIDPQMEPPMIIIEDPEQFQNGFPLFDLNNPEGENQIPLL
Prediction:     --++-++-+++----------------------------+--------+++++++-----
Confidence:     963363545227668978955975587689999998767259878879233322259789

Sequence:       QPLIDPDNQDQNNHFPPIVNGDQQDQPMENNHHNHHNFELPDFDDDNAQAGPLVNGHVPE
Prediction:     ------++++++++-----+-++++++++++++++-----------+-+--+--+-+---
Confidence:     589965234243227568834333232233342325596977977727364367363756

Sequence:       HEQLLILPPFNIDPIPPDESSSSISSSIRMSDSSGSMADDEEEYSLPDNRFLMERQSGRR
Prediction:     ---------------+--+++++++++-+++++++++-------+----+---+++++++
Confidence:     996999988959657255467673876673544525355576652757656752668499

Sequence:       PMFNQIIGDLSDNEMMVDQVDEDNENFVQEMLDADCLPVEVEIQVVPNESVNIPLCPPFP
Prediction:     +--+------+---------------------------------------+-+--------
Confidence:     445279986937679898696886788969999987659698958756537375776898

Sequence:       DFMNSDSDEEYVPRPLPTPIFNLTPRP
Prediction:     ---++++-+++--+--++---+-+-++
Confidence:     775233352337555625586473472
```

Figure S8. *In silico*-determined nucleic acid-binding potential of the *C. p. fossulana* QNU polypeptide. The DNA-interacting amino acids (left panel) and RNA-interacting amino acids (right panel) are highlighted in pink. They were predicted using the BindN server (http://www.bioinfo.ggc.org/bindn/).

```
cox2a
5’>TCAGCAGAATATTCAGGTTTTGATTctaa::ttctatagaatttgatttatttttaaataaagcacttaaagaagaacaagaaaaaaatggtaga
        agaa at  ag       t ctaa   t ta  gaat t a  tatttt aaa aaa  a t aaa a g     ga  a  a
5’>atgaaagaagataaagaaagaattactaaaaatgtaaggaatattaaatatttt:aaaaaaaaaagtaaaATATGTTTTCGATTATTAGATACTG
                                                                         cox2b
```

Figure S9. Alignment of the *Campsomeris* mtDNA sequences around the *cox2* split site. The 3’ end of *cox2a* and the 5’end of *cox2b* are highlighted yellow. Traces of 44-45 bp direct repeats are underlined. The *cox2a* termination codon (completed by polyadenylation) and *cox2b* initiation codon are shown on black background.

Supplementary tables 1-4

Table S1. Cys residue content of the COXII intermembrane domain in canonical and modified COXII polypeptides.

Eukaryotic lineage		Genus			*cox2* split into two genes	*cox2* enlarged through in-frame coding insertion or 3' terminal extension	*cox2* gene location		Number of Cys residues in the intermembrane domain of COXII, excluding two residues of the Cu_A center
							Mitochondrial genome	Nuclear genome	
Opisthokonta	Metazoa		*Homo*	29	-	-	+	-	0
		Insecta	*Drosophila*	28	-	-	+	-	0
		Insecta	*Pediculus*	27	-	-	+	-	0
		Insecta	*Scolia*	26	-	-	+	-	0
		Insecta	*Campsomeris*	3	+	-	a & b	-	3
			Venustaconcha	2	-	+[1]	+	-	4[1]
				16	-	-[2]	+	-	1
	Fungi		*Smittium*	17	-	-	+	-	1
Amoebozoa			*Vermamoeba*	30	-	-	+	-	0
Streptophyta			*Arabidopsis*	18	-	-	+	-	1
			Pisum	19	-	-	+	-	1
			Amphicarpaea	21	-	-	+	-	1
			Eriosema	31	-	-	-	+	1
			Atylosia	32	-	-	-	+	1
			Ramirezella	33	-	-	-	+	1
			Otholobium	34	-	-	-	+	1
Chlorophyta			*Chlamydomonas*		+	-	-	a & b	0
			Scenedesmus	8	+	-	a	b	3
			Polytomella	14	+	-	-	a & b	0
			Prototheca	20	-	-	+	-	1
Rhodophyta			*Wildemania*	25	-	-	+	-	1
Alveolata	Apicomplexa		*Plasmodium*	13	+	-	-	a & b	0
			Toxoplasma	10	+	-	-	a & b	1
			Theileria	15	+	-	-	a & b	0
	Dinoflagellata		*Karlodinium*	11	+	-	-	a & b	1
			Oxyrrhis	9	+	-	-	a & b	3
	Perkinsea		*Perkinsus*	12	+	-	-	a & b	1
	Ciliophora		*Oxytricha*	7	-	+[3]	+	-	3
			Paramecium	1	-	+[3]	+	-	5
			Tetrahymena	6	-	+[3]	+	-	2
Stramenopila			*Cafeteria*	22	-	-	+	-	1
			Pylaiella	4	-	+[3]	+	-	2
Rhizaria, Cercozoa			*Bigelowiella*	23	-	-	+	-	1
Excavata, Discoba, Jacobida			*Reclinomonas*	24	-	-	+	-	1

[1]M COXII has C-terminal extension of 147 amino acids; number of Cys does not include three Cys residues present in the C-terminal extension
[2]F COXII does not have C-terminal extension
[3]*cox2* is split by an insertion within a location similar to that of *Campsomeris cox2*, but is still expressed as a single, although larger polypeptide

Table S2. Relative synonymous codon usage (RSCU) by mitochondrial genes/ORFs of *C. p. fossulana*. Codon frequencies were determined using CAIcal server (http://www.genomes.urv.es/CAIcal/). Stop codons were not included in this count.

**Codon**	**Amino acid**	**RSCU**		**Codon**	**Amino acid**	**RSCU**		**Codon**	**Amino acid**	**RSCU**		**Codon**	**Amino acid**	**RSCU**	
		**mtDNA (excluding insertion)**	**3 kb insert**			**mtDNA (excluding insertion)**	**3 kb insert**			**mtDNA (excluding insertion)**	**3 kb insert**			**mtDNA (excluding insertion)**	**3 kb insert**
AAA	K	1.8	1.5	GAA	E	1.8	1.9	GAA	Q	2.0	1.9	TAA	*	-	-
AAG	K	0.2	0.5	GAG	E	0.2	0.1	GAG	Q	0.0	0.1	TAG	*	-	-
AAC	N	0.2	0.3	GAC	D	0.3	0.3	CAC	H	0.3	0.3	TAC	Y	0.2	0.2
AAT	N	1.8	1.7	GAT	D	1.7	1.7	CAT	H	1.7	1.7	TAT	Y	1.8	1.8
AGA	S	2.3	1.3	GGA	G	2.1	2.3	CGA	R	2.4	1.9	TGA	W	1.8	1.8
AGG	S	0.1	0.2	GGG	G	0.5	0.4	CGG	R	0.3	0.4	TGG	W	0.2	0.2
AGC	S	0.1	0.0	GGC	G	0.2	0.1	CGC	R	0.3	0.4	TGC	C	0.2	0.0
AGT	S	0.8	1.2	GGT	G	1.2	1.2	CGT	R	0.9	1.4	TGT	C	1.8	2.0
ACA	T	1.5	1.2	GCA	A	1.8	1.8	CCA	P	1.1	1.0	TCA	S	1.9	2.4
ACG	T	0.1	0.1	GCG	A	0.1	0.4	CCG	P	0.1	0.1	TCG	S	0.2	0.3
ACC	T	0.2	0.1	GCC	A	0.1	0.1	CCC	P	0.5	0.2	TCC	S	0.2	0.1
ACT	T	2.2	2.5	GCT	A	2.0	1.7	CCT	P	2.3	2.7	TCT	S	2.5	2.5
ATA	M	1.8	1.9	GTA	V	2.4	2.1	CTA	L	0.5	0.7	TTA	L	4.7	3.7
ATG	M	0.2	0.1	GTG	V	0.2	0.0	CTG	L	0.0	0.0	TTG	L	0.3	0.3
ATC	I	0.1	0.1	GTC	V	0.2	0.1	CTC	L	0.1	0.2	TTC	F	0.1	0.3
ATT	I	1.9	1.9	GTT	V	1.3	1.8	CTT	L	0.6	1.1	TTT	F	1.9	1.7

Table S3. Amino acid sequence similarities between *C. p. fossulana* polypeptide QNU and nucleic acid-interacting proteins.

Protein Name	Organism	Function	Alignment
Arg-Glu dipeptide repeats protein-like isoform X4	*Microplitis demolitor*<br>Metazoa<br>Hymenoptera Apocrita	DNA interacting	QNU 17 PVPDEDANMGLLLDDIDNEIDDEGGGDAVMQNHLNQNQQQGQNQQQDQNQQGQ----NQQ 72<br>P+P E A M + ++I +E D + + Q H Q QQQ Q QQQ Q QQ Q QQ<br>Sbjct 507 PLPTE-AEMSPVNEEIKSEPDLQPQNLNINQQHQQQQQQQQQQQQQQQQQQQQQQQHQQQ 565<br><br>QNU 73 GQNQQDQNQGIPNPN----NQLPPPHLNP--NNPLPHNNNLPNLQPFNP--EMLPNVPQQ 124<br>Q+QQ Q Q P P +Q P +L+ +NP+ ++P P P ++ P PQ<br>Sbjct 566 QQHQQQQQQMQPQPMINDLSQNMPRNLSQPMSNPIIIPQSIPTALPPPPPTQLPPQPPQG 625<br><br>QNU 125 VP-----QGNGHAFPDVP--FDQQID-PQ----------------MEPPMIIEDPEQFQ 160<br>+P G+ A VP Q I PQ ++PPM +P+<br>Sbjct 626 LPLNMQYSGSIQAAQTVPQNLSQNISIPQNIPQPIAQSLAASRDMIQPPMTNHAEPQPLA 685<br><br>QNU 161 NGFPL--FDLNNPEGENQI----PLLQPL--------------IDPDNQDQNNHFPPIVN 200<br>P+ +LN P+ +Q+ P QPL ++ D + F I<br>Sbjct 686 QSIPVPPLNLNIPQNLSQMSQMPPSPQPLGLTVMPTDNRINERLNDDRIPERKLFDRI-- 743<br><br>QNU 201 GDQQDQPMENN---HHNHHNFELPDFDDDNAQAGPLVNGHVPEHEQLLILPPFNIDPIPP 257<br>D+ + MENN HH+HH+ + D D P E + + P I P PP<br>Sbjct 744 ADRLPERMENNDHHHHHHHHQQQQDHQDR------------PPPEPVNLFQP--IQPPPP 789
Hypothetical protein DDB	*Dictyostelium discoideum*<br>Amoebozoa	Transcription factor	QNU 47 QNHLNQNQQQGQNQQQDQNQQGQNQQGQNQQDQNQGIPNPNNQLPPPHLNPNNPLPHNNN 106<br>Q H NQNQ Q Q+Q Q Q+QQ QNQ Q+QNQ N +Q P P P P<br>Sbjct 1198 QQHQNQNQHQHQHQHQHQHQQNQNQNQNQNQNQNQ---NQQHQQPQPQPQPQ---PQPQP 1251<br><br>QNU 107 LPNLQPFNPEMLPNVPQQVPQGNGHAFPDVPFDQQIDPQMEPPMIIEDPEQFQN--GFP 164<br>P QP P+ P QQ PQ G QQI+ P QF N +P<br>Sbjct 1252 QPQPQP-QPQPQPPQQQQQPQQQGQQPQQQQNQQQINKNNNN-----SSPLQFNNNTNYP 1305<br><br>QNU 165 LFDLNNPEGENQIPLLQPLIDPDNQDQNNHFPP--IVNGDQQDQPMENNHHNHHNFELPD 222<br>+F NN G N P L + N+ +N P V+G P +N++N++N +<br>Sbjct 1306 IF--NN--GINHQPNLPVQMQEINKPNSNFNTPTDFVSG-TSSYPFADNYNNNNNNNNNN 1360<br><br>QNU 223 FDDDNAQAGPLVNGHVPEHEQLLILPPFN--IDPIPPDESSSSISSSIRMSDSSGSMADD 280<br>+++N NG +H+Q I N + I E S++++ +D S ++ +<br>Sbjct 1361 NNNNNNNNNNYNNGAKLQHQQTNINHDTNTTLIKIETKEEIGSVNNT--NNDMSNNLQFN 1418<br><br>QNU 281 EEEYSLPDNRFLMERQSGRRPMFNQIIG 308<br>P + ++++G F+ I G<br>Sbjct 1419 PNTLPSPLLPSINKKKNGTVANFSPIYG 1446
Myb domain-containing protein	*Dictyostelium discoideum*<br>Amoebozoa	DNA binding	QNU 2 NYNHTGQYKTSNLFIPVPDEDANMGLLLDDIDNEIDDEGGGDAVMQNHLNQNQQQGNQQ 61<br>N N+ Y F P ++D+ + D+ Q Q QQQ Q QQ<br>Sbjct 372 NNNNNNNYNNITYFQPYTPFS-----IVDNSSMIVPDKQPQQQQPQQQPQQQQQQQQQQQ 426<br><br>QNU 62 QDQNQQGQNQQGQNQQDQNQ-------GIPNPNNQLPPPHLNPNNPLPHNNNLPNLQPFN 114<br>Q Q QQ Q QQ Q QQ Q Q N NN + N NN +NNN N+ N<br>Sbjct 427 QQQQQQQQQQQQQQQQQQQQQNYNDDSNKNNNNNNNNNNNNNNNNNNNNNNNNENINSSN 486<br><br>QNU 115 PEMLPNV------------PQQVPQGNGHAFPDVPFDQQID-PQMEP--PMIIEDPEQF 159<br>+ N+ +Q + P PF+ + PQ +P P P<br>Sbjct 487 NNNMYNICPAAAYQNIQFIKEQSNSSLSSSQPIPPFNLYNEQPQQQPQQPQPTQSQPILS 546<br><br>QNU 160 QNGFPLFDLNNPEGENQIPLLQPLIDPDNQDQNNHFPPIVNGDQQDQPMENNHHNHHNFE 219<br>+ +FD+N+ Q Q Q Q QQ Q + N<br>Sbjct 547 SSSTSVFDINHHHHHQQQQQQQQQQQQQQQQQQ----------QQQQQQQQQQQPQPNLS 596<br><br>QNU 220 LPDFDDDNAQAGPLVNGHVPEHEQLLILPPFNIDPIPPDESSSSISSS--IRMSDSSGSM 277<br>+ D+N + +G+V E + I I PP + SSI+ + + +S S+ S+<br>Sbjct 597 SSSYADNN-NSFQSSSGNVWESQSSPIQSSVQISS-PPQSNQSSIAPAPAVNLSASASSV 654<br><br>QNU 278 ADDEEEYSLPDNRFLMERQSGRRPMFNQIIGDL---SDNEMMVDQVDEDNENFVQEMLDA 334

			A ++ ++ + ++Q ++ Q + D +DN +D+ DED++ +M D<br>Sbjct 655 ATTTKQSNVKKQKQQQQQQQQQQQTKRQELSDSEDDTDNGDDIDEDDEDDDEDEDDMEDE 714<br>QNU 335 D 335<br>D<br>Sbjct 715 D 715
Mediator of RNA pol II transcription subunit 15 isoform c	*Homo sapiens*<br>Metazoa	Transcription	QNU 47 QNHL----NQNQQQGQNQQQD-------QNQQGQNQQGQNQQDQNQGI-PNPNNQLPP-- 92<br>Q HL +QNQQQ Q QQQ Q QQ Q QQ Q QQ Q Q + P Q PP<br>Sbjct 217 QQHLIKLHHQNQQQIQQQQQQLQRIAQLQLQQQQQQQQQQQQQQQQALQAQPPIQQPPMQ 276<br><br>QNU 93 -PHLNPNNPLPH--------NNNLPNLQPFNPEMLPNVPQQVPQGNGHAFPDVPFDQQID 143<br>P P+ LP ++ P QP P + N P Q+P P V Q +<br>Sbjct 277 QPQPPPSQALPQQLQQMHHTQHHQPPPQPQQPPVAQNQPSQLPP-QSQTQPLVSQAQALP 335<br><br>QNU 144 PQM---EPPMIIIEDPEQFQNGFPLFDLNNPEGENQIPLLQ-----PLIDPDNQDQNNHF 195<br>QM +PP+ + P Q P+ P+ + Q +Q ++ P Q Q<br>Sbjct 336 GQMLYTQPPLKFVRAPMVVQQP-PV----QPQVQQQQTAVQTAQAAQMVAPGVQVQTPQS 390<br><br>QNU 196 --PPIVNGDQQDQPMENNHHNHHNFELPDFDDDNAQAGPLVNGHVPEHEQLLILPPFNID 253<br>PP Q QP + N + P P P + P N<br>Sbjct 391 MPPPPQPSPQPGQPSSQPNSNVSSGPAPSPSSFLPSPSP-----QPSQSPVTARTPQNFS 445<br><br>QNU 254 PIPPDESSSSISSSIRMSDSSGSMADDEEEYSLPDNRFLMERQSGRRPMFNQI------I 307<br>P ++ ++ S MS + S A EE+ L + L + R M N+I<br>Sbjct 446 VPSPGPLNTPVNPSSVMSPAGSSQA--EEQQYLDKLKQLSKYIEPLRRMINKIDKNEDRK 503<br><br>QNU 308 GDLSDNEMMVDQVDEDNENFVQEMLDADCLPVEVEIQVVPNE-SVNIPLCPPFPDFMNSD 366<br>DLS + ++D + + ++ + L C E+ ++ + N+ +V P PP P<br>Sbjct 504 KDLSKMKSLLDILTDPSKRCPLKTLQ-KC---EIALEKLKNDMAVPTPPPPPVP-----P 554<br><br>QNU 367 SDEEYVPRPL--------PTPIFN 382<br>+ ++Y+ +PL +P+FN<br>Sbjct 555 TKQQYLCQPLLDAVLANIRSPVFN 578
Heat shock factor-type DNA-binding domain-containing protein	*Dictyostelium discoideum*<br>Amoebozoa	DNA binding	QNU 11 TSNLFIPVPDEDANMGLLLDDI------DNEIDDEGGGDAVMQNHLNQNQQQGQNQQQDQ 64<br>T++ F P + L++DI +N ++ + N+ N +QQQ Q QQQ Q<br>Sbjct 271 TNSQFNPQNNLQNKQNNLVNDIISTNNNNNNNNNNNSNNNNNNNNNNNSQQQQQQQQQQ 330<br><br>QNU 65 NQQGQNQQGQNQQDQNQGIPNPNNQLP-PPHLNPNNPLPHNNNLPNLQPFNPE-MLPNVP 122<br>QQ Q QQ Q Q Q Q P P +P PP L P H +QP P P P<br>Sbjct 331 QQQQQQQQQQQHQIQMQPTP-PQLPMPTPPQLPMPTPPQH-----QMQPTPPSPQQPTPP 384<br><br>QNU 123 -QQVPQGNGHAFPDVPFDQQIDPQMEPPMIIIEDPEQFQNGFPLFDLNNPEGENQIPLLQ 181<br>QQVPQ P P QQ Q + II P P N G NQ<br>Sbjct 385 QQQVPQSQ---IPSSPQQQQ---QQQQQQYIISPPSY----SPTSSSNQIGGFNQ----- 429<br><br>QNU 182 PLIDPDNQDQN--NHFPPIVNGDQQDQPMENNHHNHHN 217<br>I+ N N N +NG+ + NN++N++N<br>Sbjct 430 --INKSNGSTNSINSNNSTINGNNNNNSNNNNNNNNNN 465
Putative protein associated with topo II-related 1	*Danaus plexippus*<br>Metazoa<br>Lepidoptera	Telomere extension	QNU 2 NYNHTGQYKTSNLFIPVPDEDANMGLLLDDIDNEIDDEGGG---DAVMQN---HLNQNQQ 55<br>N N Q++ + + + ++ NM L + +N++ G + ++QN ++NQ Q<br>Sbjct 260 NSNQMNQFQNNQMAPAMMNQMHNMNLHPMNQNNQMIQNQMGQNMNQMVQNSGSNMNQMNQ 319<br><br>QNU 56 QGQNQQQ---DQNQQGQN--QQGQN--QQDQNQGIPNPN----NQL-----------PPP 93<br>GQN Q + NQ GQN Q GQN Q QNQ IPN N NQ PPP<br>Sbjct 320 GGQNMNQMGQNMNQMGQNMNQMGQNMNQMGQNQMIPNGNQFGMNQFQQMMGQPRMMAPPP 379<br><br>QNU 94 HLNPNNPLPHNNNLPNLQPFNP----EMLPNVPQQVPQGNGHAFPDVPFDQ----QIDPQ 145<br>+N +P N N+ FN +M QQ+ Q N + +Q Q+ P+<br>Sbjct 380 GMN----MPRQNFGNNMNQFNTNFRGQMQSKNDQQISQQNMIMNHNKNQNQVKPNQLSPK 435<br><br>QNU 146 MEPPMII--IEDPEQFQNG----FPLFDLNNPEGENQIPLLQ---PLIDPDNQDQNNHFP 196<br>+P + I++ Q QN +++ + +N + L+Q P+++ +N N<br>Sbjct 436 QKPVQNLNHIQNMNQAQNNKLQKSRVYNNKSLTSQNLVQLIQNTHPMLNFNNSFHNASHH 495<br><br>QNU 197 PIVNGDQ-QDQPMENNHHNHHNFELPDFDDDNAQAGPLVNGHVPEHEQLL 245<br>PI+N + +Q M+ H N + +F + ++A V+G + E+ L+<br>Sbjct 496 PILNRNHFNNQLMK--HLMFDNRQNGNFGNTTSRANTSVDGELDEYAGLM 543

Transcription factor SPT20 homolog	*Nasonia vitripennis*<br>Metazoa<br>Hymenoptera Apocrita	Transcription factor	QNU 47 QNHLNQNQQQGQNQQQDQNQQGQNQQGQNQQDQNQGIPNPNNQLPPPHLNPNNPLPHNNN 106<br>Q H Q QQ Q QQ Q QQ Q QQ Q Q Q Q P Q PP P +<br>Sbjct 467 QQHQQQQHQQQQQQQLQQQQQHQQQQQQQHQQQQQQSQMPGQQQGPPQQQQGAPQQQQGS 526<br><br>QNU 107 L-PNLQPFNPEML--PNVPQQVPQGNGHAFPDVPFDQQIDPQMEPPMIIIEDPEQFQNGF 163<br>+ P Q P+ P PQQ PQ P P Q PQ + P Q Q<br>Sbjct 527 MPPPSQQGGPQQQQPPQQPQQQPQQQPQQQPPHP-PQMPSPQSQMP------GGQNQQQM 579<br>QNU 164 PLFDLNNPEGENQIPLLQPLIDPDNQDQ--NNHFPPIVNGDQQDQPMENNHHNHH----- 216<br>P P+ + P P P +Q Q ++ PP ++ QQ Q + H<br>Sbjct 580 P------PQPQQGQPPQMP-TGPSSQQQPMSSQQPPQMSPQQQQQQQQQQQQQQHMPPQQ 632<br><br>QNU 217 NFELPDFDDDNAQAGPLVNGHVPEHEQLLILPPFNIDPIPPDESSSSISS 266<br>++P A P VP H+Q + P +P +S+++ ++<br>Sbjct 633 QQQMPPQQQLGA-GSPYPQQQVPPHQQAIPGGPPQPGKMPQSQSTTAAAA 681
SNF2-family ATP dependent chromatin remodeling factor snf21	*Candida tropicalis*<br>Fungi	Chromatin remodeling	QNU 45 VMQNHLNQNQQQGQNQQQDQNQ---QGQN------QQGQNQQDQNQGIPNP--------- 86<br>+ Q Q QQQ Q QQQ Q Q QG N QQ Q Q Q Q P P<br>Sbjct 46 MQQQRFQQQQQQQQQQQQQQQQQQRQGMNSQSSMPQQMQTQTPQQQQSPAPQQFTNMNQN 105<br><br>QNU 87 --NN-----QLPPPHL------NPNNPLPHNNNLP---------NLQPFNPEM-LPNVPQ 123<br>NN Q+ P + N PLP N N+ N FN +M + + Q<br>Sbjct 106 GFNNQPYQSQMHSPAIGGSLSNNGTPPLPQNTNMMANSNKNMKLNSNQFNNQMGMSPMQQ 165<br><br>QNU 124 QVPQGNGHAFPDVPFDQQIDPQMEPPM----IIIEDPE------QFQ------NGFP-LF 166<br>Q + P F+Q QM+PP +PE QFQ F L<br>Sbjct 166 QSQYSSQSPNPSNMFNQMSQQQMQPPQHQQSSTTPNPESAFTNQQFQLLKSQLQAFKYLV 225<br><br>QNU 167 DLNNPEGENQIP--LLQPLIDPDNQDQNNHFPPIVNGDQQDQPMENNHHNHHNFELPDFD 224<br>G QIP L+ + +P + N+ + P VN Q + M+ + +LP +<br>Sbjct 226 KAPGQGGPGQIPQNLIAYVSNPSSAMANDMYLPAVNRPQSNGMMQMPINQQMGQQLPP-N 284<br><br>QNU 225 DDNAQAGPLVNGHVPEHEQLLILPPFNIDPIPPDESSSSISSSIRMSD-SSGSMADDEEE 283<br>Q PL P +Q P P D S++S+ + M + G ++<br>Sbjct 285 MQQQQQPPL----PPPPQQQQQFP----GQQPNDMSNNSVGGTPEMGEKKKGKRGPRPKQ 336<br><br>QNU 284 YSLPDNRFLMERQSGRRPMFNQIIGDLSDNEMMVDQVDEDNENFVQEMLDADCLPVEVEI 343<br>P + L E + +R + +L N + +N + + PV+ +<br>Sbjct 337 PKKPTKKQLREEE--QRLALERQRQELEQNRL---------KNDISQAFPGGPFPVQSQF 385<br><br>QNU 344 QVVPNES------VNIPLCPPFPDFMNSDSDEEYVPRPLPTP 379<br>P + +P PP P + VP P+P P<br>Sbjct 386 TPQPQQQQQQQQPPQVPGQPPLPQQRQMSMGPQSVPGPIPPP 427
RNA splicing factor 3B subunit 4	*Dictyostelium discoideum*<br>Amoebozoa	RNA splicing	QNU 54 QQQGQNQQQDQNQQGQNQQGQNQQDQNQGIPNPNNQLPPPHLNPNNPLPH-NNNLPNLQP 112<br>QQQ Q QQQ Q QQ Q QQ Q QQ Q Q P+ + +P PH P+ PH + + P P<br>Sbjct 4275 QQQQQQQQQQQQQQQQQQQQQQQQQQQQHHPHHQHPIPHPHPLPHQLRPHPHPHHPPPPP 334<br><br>QNU 113 FNPEMLPNVPQQVPQG 128<br>FNP ++ P +P G<br>Sbjct 335 FNPMLMQFNPMMMPFG 350

Table S4. Primers used in RT-qPCR.

Organism	Transcript	Forward primer	Reverse primer
D. melanogaster	*cox1*, *cox1* complement	CTACTACCTCCTGCTCTTTCTTTAC	TGAGCAATTCCAGCGGATAA
	cox2, *cox2* complement	CGATTATTAGATGTTGATAACCGAGTAG	AGCAGGTACTGTTCAAGAATGA
	nad4, *nad4* complement	CCCAGAAGAACATAAACCATGAG	CATCTGTTGCTCATATAGGAATTGT
	atp6, *atp*6 complement	ATATATTTGCTCATTTAGTTCCTCAAG	CGAACAGCTAATGTTCCAGGT
	cob	TGAGGTGGATTTGCTGTTGATA	GGATTATTAGATCCTGTTTGATGAAGG
C. p. fossulana	*cox1*, *cox1* complement	CCTGATATAGCTTTCCCTCGATTA	TAGTTCATCCTGTCCCAATTCC
	cox2a, *cox2a* complement	AACTCACGGTAGATTTATCGAAG	ATACTAGGATTTAATGTACCTTCATCA
	cox2b, *cox2b* complement	GTTGTACCAATCAATAACCCTATTCG	GGAATTCTGTCTACCTTTACTCCTATT
	qnu, *wfw*	GGATCTATAGCTGATGACGAAGAAG	GTTGTCTCTGAGATCGCCAATAA
	orf3, *orf6*	CCTCCTCCAATAAATACCGATAGAG	GGCCTAGGACTATCGTCTATATCTAA
	orf4, *orf7*	TCAATAGTTTGTAATCCACTTCCAC	ATTAATATAGAAGACGACCCTCCTAC
	orf5, *orf9*	CACTATTACTAGAAGATGGGCCATAA	GAACATACTTCACCATGTAATAGTTCAC
	orf8	GAACTATTACATGGTGAAGTATGTTCAG	CTCCTACTCCTTCTAAACCAACAA
	nad4, *nad4* complement	AAGAGGGATATTAGGTGCTCAAAT	AACCGTCTTCTAGTACGTTCATAAT
	atp6, *atp6* complement	AAATGGCCAGCCGTTAAATTAG	GCTCATTATGTTCCAACATCCAC
	cob	GTTGCACCTCATAAAGATATTTGACC	GCACATGAGCAGTAGGTGTTAT
